# Wnt-inducible Lrp6-APEX2 interacting proteins identify ESCRT machinery and Trk-fused gene as components of the Wnt signaling pathway

**DOI:** 10.1038/s41598-020-78019-5

**Published:** 2020-12-09

**Authors:** Gabriele Colozza, Yasaman Jami-Alahmadi, Alyssa Dsouza, Nydia Tejeda-Muñoz, Lauren V. Albrecht, Eric A. Sosa, James A. Wohlschlegel, Edward M. De Robertis

**Affiliations:** 1 Department of Biological Chemistry, David Geffen School of Medicine, University of California, Los Angeles, USA; 2 Howard Hughes Medical Institute, University of California, Los Angeles, CA 90095-1662 USA; 3 Present Address: Institute of Molecular Biotechnology of the Austrian Academy of Sciences (IMBA), Vienna Biocenter (VBC), Vienna, 1030 Austria; 4 Department of Genetics, Albert Einstein College of Medicine, 1300 Morris Park Ave., Bronx, NY 10461 USA

**Keywords:** Developmental biology, Cell biology, Cell signalling

## Abstract

The canonical Wnt pathway serves as a hub connecting diverse cellular processes, including β-catenin signaling, differentiation, growth, protein stability, macropinocytosis, and nutrient acquisition in lysosomes. We have proposed that sequestration of β-catenin destruction complex components in multivesicular bodies (MVBs) is required for sustained canonical Wnt signaling. In this study, we investigated the events that follow activation of the canonical Wnt receptor Lrp6 using an APEX2-mediated proximity labeling approach. The Wnt co-receptor Lrp6 was fused to APEX2 and used to biotinylate targets that are recruited near the receptor during Wnt signaling at different time periods. Lrp6 proximity targets were identified by mass spectrometry, and revealed that many endosomal proteins interacted with Lrp6 within 5 min of Wnt3a treatment. Interestingly, we found that Trk-fused gene (TFG), previously known to regulate the cell secretory pathway and to be rearranged in thyroid and lung cancers, was strongly enriched in the proximity of Lrp6. TFG depletion with siRNA, or knock-out with CRISPR/Cas9, significantly reduced Wnt/β-catenin signaling in cell culture. In vivo, studies in the *Xenopus* system showed that TFG is required for endogenous Wnt-dependent embryonic patterning. The results suggest that the multivesicular endosomal machinery and the novel player TFG have important roles in Wnt signaling.

## Introduction

The Wnt/β-catenin pathway is an evolutionarily conserved signaling cascade that plays a fundamental role in animal embryonic development, adult stem cell homeostasis, and is dysregulated in a number of human diseases, including cancer^1,2^. In the absence of Wnt stimulation, the scaffold proteins Axin and Adenomatous Polyposis Coli (APC) form a ‘destruction complex’ with Casein Kinase1 (CK1) and Glycogen Synthase Kinase 3β (GSK-3β) which sequentially phosphorylate β-catenin and mark it for ubiquitination and proteasome-mediated degradation^3^. Canonical Wnt signal transduction is initiated when a Wnt ligand binds to the seven-pass transmembrane receptor Frizzled (Fzd) and the Low-density lipoprotein receptor-related protein 5/6 (Lrp5/6). The activated co-receptors recruit a number of effectors, including CK1 isoforms^4^, the adaptor Dishevelled (Dvl), Axin1 and GSK-3β, to the cytosolic side of the plasma membrane forming a complex known as the signalosome^5^. Signaling requires specific protein–protein interactions and the phosphorylation of several components, including the cytoplasmic tail of Lrp6^6–9^. Subsequently, activated Wnt receptor clusters^10^ are internalized into multivesicular bodies by Endocytic Sorting Components Required for Transport (ESCRT)-driven microautophagy of GSK-3β and other negative Wnt regulators such as Axin, sequestering them away from their cytosolic targets^11,12^. As a result, β-catenin protein is stabilized and translocates into the nucleus, where it binds to T-cell factor/lymphoid enhancer-binding factor (TCF/LEF) DNA-binding proteins to regulate the transcription of Wnt-target genes.

During the 30 years since its discovery^13,14^, the Wnt pathway has been the subject of intense studies, but its molecular mechanisms have not yet been fully elucidated, in particular the role of membrane trafficking in receptor activation^11,12,15–17^. To dissect the dynamics of Wnt signaling, we decided to use a protein interaction approach to identify additional regulators of the pathway. In recent years, proximity-dependent biotin labeling approaches have emerged as a valuable tool to define functional protein complexes or organelle-specific proteomes^18^. Two classes of enzymes can be used in proximity labeling (PL) experiments: the promiscuous *E. coli* biotin ligase BirA R118G (BirA*)^19^ and its variants TurboID and miniTurboID^20^, or the engineered soybean Ascorbate Peroxidase 2 (APEX2)^21^. These enzymes attach biotin-derivatives covalently to nearby proteins in vivo, while still in their native cellular environment. Biotinylated targets can then be extracted, purified by streptavidin-bead pull-down, and identified by mass spectrometry (MS). The APEX2 peroxidase technology offers several advantages: faster kinetics, robust biotinylation activity, and the possibility to activate the labeling at specific moments by adding H_2_O_2_ to living cells^21,22^. APEX2 requires the use of biotin-phenol (also known as biotin-tyramide), which in combination with H_2_O_2_ is converted to a highly reactive, short-lived (< 1 ms) radical with a labeling radius of 20 nm. The high spatial and temporal specificity provided by APEX2 has allowed the study of the proteome of organelles or subcellular compartments difficult to isolate, such as the mitochondrial intermembrane space and endoplasmic reticulum (ER) membrane^23^, and to study G-protein-coupled receptor (GPCR) signaling in great detail^24,25^.

Here, we describe the results obtained with a Human Embryonic Kidney 293 T (HEK293T) cell line stably expressing a chimeric Lrp6-APEX2 receptor. Our proteomic data indicate that in presence of Wnt3a, the Lrp6-interactome was enriched in components of the ESCRT machinery that forms MVBs, providing independent support to our previous findings that endocytosis is at the core of Wnt pathway intracellular regulation^11,15,16^. We also discovered that Tropomyosin-receptor kinase fused gene (Trk-fused gene, TFG), a protein involved in several pathologies including cancer^26^, was a highly enriched target of Wnt-activated Lrp6-APEX2 and localized to endosomal vesicles. Cell culture knock-out assays and in vivo experiments using *Xenopus laevis* embryos revealed an important role for TFG in Wnt/β-catenin signaling and antero-posterior (AP) patterning.

## Results

### Lrp6-APEX2 receptor fusion is active in Wnt signaling

**Figure 1:**
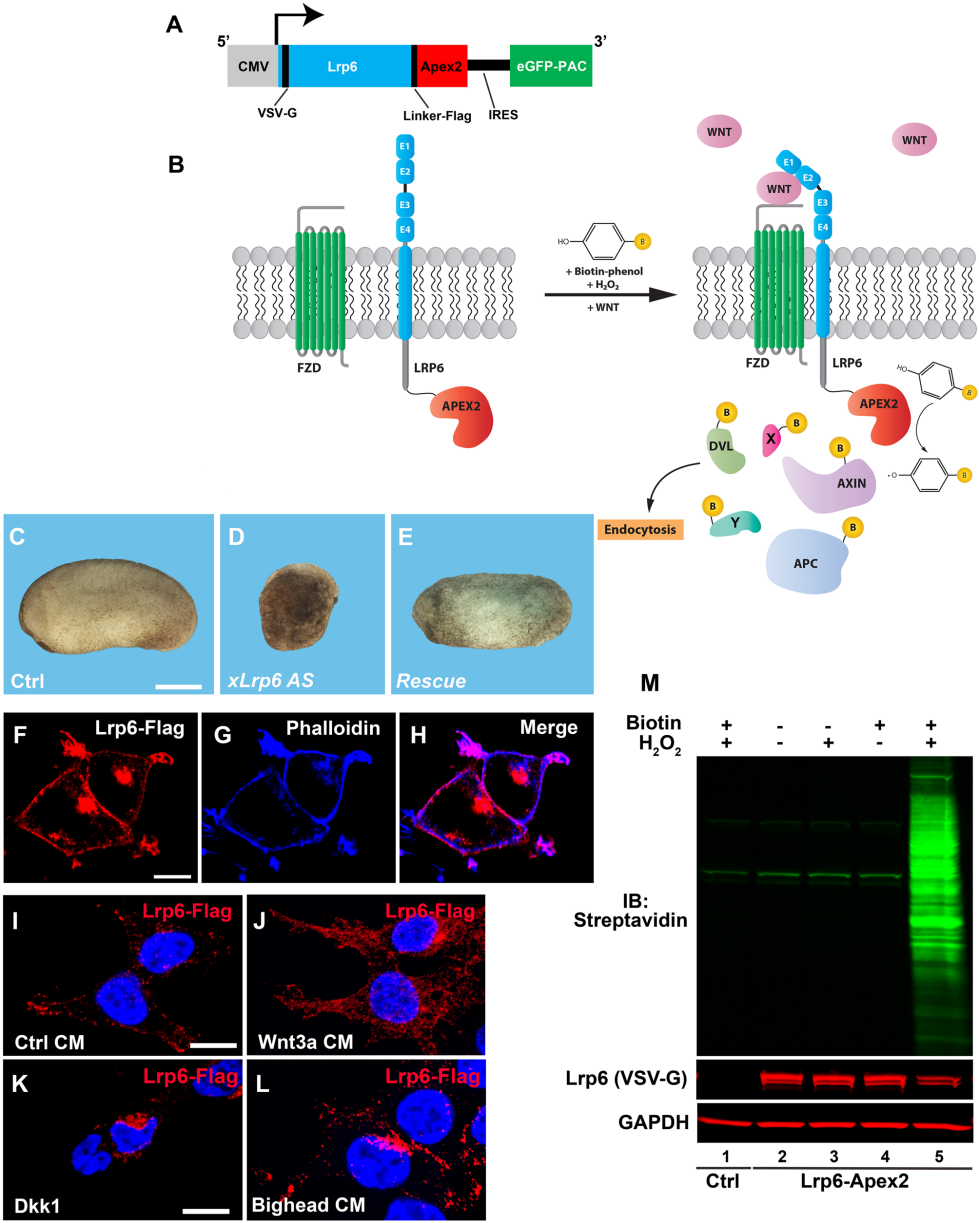
The Lrp6-APEX2 fusion protein receptor retains signal transducing activity, proper subcellular localization, and biotinylates cellular proteins. (**A**) Schematic diagram of the Lrp6-APEX2 construct. The human Lrp6 contains an N-terminal VSV-G and a linker Flag-tag followed by the APEX2 enzyme. The construct also contains an internal ribosomal entry site (IRES) followed by a GFP-tagged Puromycin N-acetyltransferase (PAC) used for selection of permanently transfected cells. (**B**) Diagram depicting the biotinylation of Lrp6-APEX2 proximal targets. During the Wnt signal, in the presence of Biotin-phenol (BP) and H_2_O_2_, proteins recruited to the receptor are biotinylated by the APEX2 peroxidase, including known Wnt targets (Dvl, Axin, APC, etc.) and novel targets (X, Y). (**C–E**) control embryos from oocyte-host transfer experiments show normal development (100%, n = 24), as compared to oocytes depleted with a phosphorothioate antisense DNA oligo targeting *Xenopus* Lrp6^27^, which develop as ventralized embryos (75%, n = 22). Co-injection of 300 pg of Lrp6-APEX2 mRNA into oocytes completely rescued axis formation (60%, n = 17), indicating that the fusion protein is fully active. Scale bar represents 500 µm. (**F–H**) Lrp6-APEX2 is trafficked to the plasma membrane in stably transfected HEK293T cells. Lrp6 was detected through its Flag-tag and Phalloidin, which stains cortical F-Actin, confirmed cell surface localization of Lrp6. Scale bar represents 10 µm. (**I**–**L**) Lrp6-APEX2 changes subcellular localization by Wnt, Dkk1 and Bighead treatments. In presence of control conditioned medium (CM), Lrp6 is located mostly at the plasma membrane (79%). Treatment with Wnt3a CM for 30 min increases the number of intracellular vesicles containing Lrp6 (53%). Treatment with Dkk1 protein (200 ng/ml) or Bighead CM induced relocation of Lrp6 to the juxtanuclear bay area where lysosomes are located (63% and 53%, respectively). Quantification was obtained counting cells showing the observed immunostaining over total nuclei, in 10 different micrographs. Scale bars represent 10 µm. (**M**) western blot stained with Streptavidin-IRDye 800 (in green) showing controls indicating that Lrp6-APEX2 biotinylates proximal proteins only in the presence of both BP and H_2_O_2_ (compare lane 5 with 2–4). Negative control cells not expressing the APEX2 peroxidase had no biotinylation even in presence of BP/H_2_O_2_ (lane 1). Note the presence of three Streptavidin-positive bands in all lanes, which correspond to endogenous biotinylated carboxylases. Gapdh served as a loading control.

To study the protein complexes that are recruited in proximity of the receptor complex during Wnt signaling, we appended APEX2^21^ to the cytoplasmic tail of the Lrp6 receptor (Fig. 1A,B). The chimeric receptor maintained functionality, since mRNA overexpression could rescue the ventralized phenotype caused by maternal depletion of *Xenopus* Lrp6^27^ in frog embryos (Fig. 1C–E), and induced axis duplication in a ligand dependent manner when overexpressed in a ventral blastomere (Fig. S1A–G), as is the case with the wild type receptor^6^. Importantly, Lrp6-APEX2 also restored the response to Wnt ligands by Lrp5/6 double knock-out HEK293 cells^28^, corroborating its functionality (Fig. S1H). Next, we established a HEK293T stable clonal line expressing the Lrp6-APEX2 construct, which included the GFP-tagged puromycin selectable marker eGFP-PAC after an internal ribosome entry site (IRES) (Fig. 1A). Lrp6-APEX2 was robustly expressed (Fig. S1I), and was properly trafficked to the plasma membrane (Fig. 1F–H). Treatment with Wnt3a conditioned medium translocated Flag-tagged Lrp6-APEX2 into cytoplasmic vesicles within 30 min (compare Fig. 1I–J).

Wnt activation is known to induce endocytosis of the receptor complex^11,12^. We confirmed that Lrp6 endocytosis was observed in SW480, a colon cancer cell line in which the absence of APC triggered, and APC reconstitution inhibited, Wnt signaling and Lrp6 endocytosis (Supplementary Movie S1); this is in agreement with recent results showing that APC is a major regulator of endocytosis^16,29^. As expected for regulators of Lrp6 endocytosis, the Wnt antagonists Dkk1^30^ or Bighead^31^ induced endocytosis of Lrp6-APEX2 into large vesicle clusters in the cytoplasm next to the nuclear bay region, where the lysosomal system is located (Fig. 1K,L). Next, we assessed the activity of the APEX2 enzyme. The western blot in Fig. 1M includes several control treatments showing that APEX2-mediated biotinylation only occurred in the presence of both biotin-phenol and H_2_O_2_. This activity could also be detected by Streptavidin immunofluorescence (Fig. S1J–O). Altogether, our data show that the chimeric receptor Lrp6-APEX2 can replace the wild type receptor and can be used for biotin-labeling studies.

Compared to the parental HEK293T cell line, Lrp6-APEX2 HEK293T cells showed elevated levels of phosphorylated Dvl2 (pDvl2) (Fig. S2A); however, this Dvl2 phosphorylation was blocked upon preincubation with the Porcupine inhibitor IWP-2^32^, indicating a higher sensitivity to an unknown endogenous Wnt present in HEK293T cells (Fig. S2B). In the presence of IWP-2, high levels of phospho-Dvl2, active (i.e., non-phosphorylated) β-catenin and phospho-Lrp6 (pLrp6) were only observed when cells were exposed to Wnt3a conditioned medium and, interestingly, this response was increased by R-Spondin 1 and 2 (Fig. S2C). Thus, our Lrp6-APEX2 cells are entirely dependent on exogenous Wnt extracellular ligands, providing a new and sensitive system to explore the interactions of the Lrp6 receptor during Wnt signaling.

### Wnt induces Lrp6-APEX2 biotinylation of components of the ESCRT machinery

**Figure 2:**
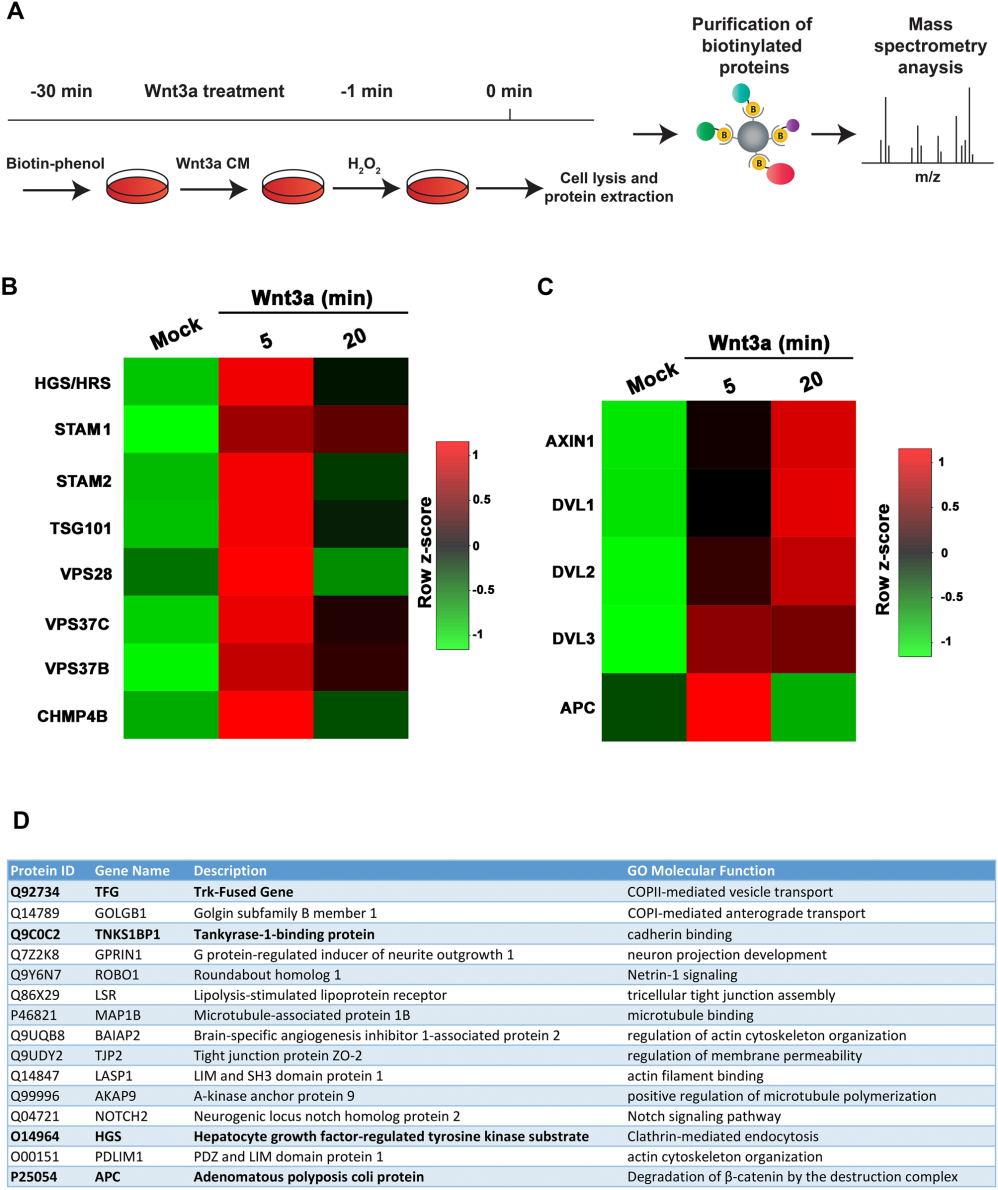
Analysis of Lrp6-APEX2 proximal targets reveal rapid Wnt3a-induced interaction between Lrp6 and ESCRT proteins. (**A**) Schematic of the biotinylation proteomic experiments reported in this study. (**B**) Heatmap of Lrp6-APEX2 proximity labeled ESCRT proteins using normalized intensities at 5 and 20 min time points, showing the horizontal row z-scores of proteins over time. Mock indicates cells treated with control conditioned medium from cells not expressing Wnt3a. Row z-scores were calculated from average intensities of proteins. Average intensity values were obtained from biological duplicates. Highly biotinylated proteins are shown in red and lower ones in green. Note the enrichment in ESCRT proteins particularly after 5 min of Wnt3a treatment. (**C**) Heatmap of Lrp6-APEX2 proximity labeled known canonical Wnt signaling pathway target proteins using normalized intensities at 5- and 20-min time points, showing the horizontal row z-scores of proteins over time. Average intensity values were obtained from biological duplicates. Highly biotinylated proteins are shown in red and lower ones in green. Note the interaction between Lrp6-APEX2 and Dvl 1–3 and Axin proteins after 20 min of Wnt3a treatment while APC peaks at 5 min; these interactions support the specificity of our Wnt3a signaling experiments. (**D**) The top 15 biotinylated targets (ranked according to their spectral counts) from Supplementary Table S1 Tab 1. Note the presence of the ESCRT-0 protein Hrs/Hgs, the Wnt inhibitor APC and Actin remodeling proteins such as BAIAP2, LASP1 and PDLIM1. Unexpectedly, the Trk-fused gene (TFG) was the most enriched protein in this list.

Lrp6-APEX2 HEK293T cells (preincubated with Porcupine inhibitor to eliminate endogenous Wnts) were treated for 30 min with biotin-phenol in T225 flasks (Fig. 2A). Next, duplicate samples were treated with either control or Wnt3a conditioned medium containing biotin-phenol (fortified by the addition of 50 ng/ml R-Spo1 and 2 which were found to strongly increase Wnt3a responsiveness; Fig. S2D). Two time points were analyzed, 5 and 20 min. The labeling reaction was triggered by a 1-min incubation with H_2_O_2_, followed by cell lysis and pull-down of biotinylated proteins (Fig. 2A). Each treatment included biological duplicates. Biotinylation and β-catenin stabilization were confirmed by western blot in each sample (Fig. S2E). Affinity-purified samples were then processed for tandem mass spectrometry (MS/MS) of tryptic peptides^33,34^ as described in “Methods”. All original datasets generated in this study are publicly available at the MassIVE public repository resource (accession number MSV000084335).

Principal Component Analysis (PCA) showed that each replicate clustered according to the type of treatment, validating the reproducibility of the assay (Fig. S2F). Analysis of the peptide data generated a list of over 4,000 putative biotinylated proteins (all of which are listed in Tab 1 of Supplementary Table S1). Of these, over 2,000 could be accurately quantified using MS1-based label free quantitation (Supplementary Table S1, Tab 2), as explained in “Methods”. Of those 2,000-plus proteins, 217 and 237 proteins were significantly enriched (p-value < 0.05) in the 20 and 5 min Wnt3a samples, respectively, compared to no biotin controls (Supplementary Table S1, Tab 3). Amongst the most highly enriched proteins was the Trk-Fused Gene (TFG). The full list of identified proteins (including their spectral counts) that interact with Lrp6-APEX2 after 5 or 20 min of Wnt3a treatment is shown in Supplementary Table S1 and represents a rich resource to search for downstream interactions of the Lrp6 receptor during Wnt signaling.

Gene Ontology analyses using the Panther database revealed that the top hit induced by Wnt3a conditioned medium was the ESCRT machinery required for MVB formation. Heatmaps derived from the normalized list of proteins showed a clear involvement of the ESCRT machinery, with a marked enrichment of Hrs, Stam1/2, Chmp4, Vps28, Vps35b, Vps35c and Tsg101 among others, which was strongest after 5 min of Wnt3a treatment (Fig. 2B). Confirming that the Lrp6-APEX2 interactions were Wnt-induced, known targets of the canonical Wnt pathway, such as APC, Dvl 1–3 and Axin and were biotinylated upon Wnt3a treatment, although in this case the interactions at 20 min were maximal, except for APC which was maximal at 5 min (Fig. 2C). We were surprised not to detect some known Lrp6 interactors, like Frizzled. However, it should be noted that APEX-mediated biotinylation depends on the availability of certain amino acid residues, like tyrosine^21,22^, on the target protein and factors like steric hindrance and accessibility may affect efficient labeling. It is possible that insufficient biotinylation precluded Frizzled detection on our proteomic analysis.

Proteins involved in actin cytoskeleton remodeling were also prominent among Wnt-inducible interactions. Since canonical Wnt signaling triggers macropinocytosis through the actin contractile machinery, this might be indicative of a role of Lrp6 in plasma membrane macropinocytosis^16,35^. Figure 2D shows a partial list including the 15-most differentially enriched biotinylated proteins upon a 20 min Wnt3a treatment, which included TFG, Tankyrase-1 binding protein, Hrs/Hgs and APC.

We also carried out proteomic analysis of a single sample treated for 1 h with Wnt3a conditioned medium (CM) or CM from control cells not expressing Wnt3a, designated Mock CM (Supplementary Table S1, Tab 4). This longer time point (which did not include the use of R-spondin 1 and 2) revealed that the top three Lrp6-APEX2 interactors were α-Spectrin, β-Spectrin and their ancestor α-Actinin (from which spectrins evolved)^36^. These proteins form a meshwork at the plasma membrane (together with protein band 4.1) that serves to link actin filaments to the plasma membrane^36^. The continuous involvement of the actin cytoskeletal machinery is consistent with a sustained role for nutrient acquisition via macropinocytosis in canonical Wnt signaling, which depends on Gsk3 inhibition^16,37,38^. We also performed MS analysis on whole protein extracts derived from the 1 h Wnt3a treatment, before any streptavidin-based enrichment, confirming the stabilization of β-catenin by Wnt (Supplementary Table S1, Tab 5).

We next analyzed more deeply this global proteomic data using the Metascape online software. Wnt3a-induced biotinylated proteins were ranked for enrichment over Mock control CM samples. The lists of the top 150 proteins were analyzed for Gene Ontology groups at 5 and 20 min and 1 h, as shown in Fig. S3A–C. After 5 min endocytosis was the top hit, followed by small GTPase-mediated signal transduction. Interestingly, Kras was notable among Wnt-induced Lrp6 interacting proteins, both after 5 and 20 min. This is important because Kras is a major regulator of many metabolic processes, including actin-driven macropinocytosis. In the 20 min sample, the top GO score was signaling by Wnt, as might have been expected. After 1 h of Wnt, actin cytoskeleton organization and membrane trafficking were pre-eminent.

In summary, our searchable list of Lrp6-APEX2 biotinylated targets (Supplementary Table S1, Tab 1 and Tab 4) offers the scientific community a valuable roadmap for the discovery of novel proteins involved in Wnt signaling and endocytosis. Importantly, it independently confirms our earlier proposals^11^ that the ESCRT machinery and endocytosis^15, 16^ participate in Wnt signaling.

### TFG localizes to endocytic vesicles and is required for Wnt signaling

**Figure 3:**
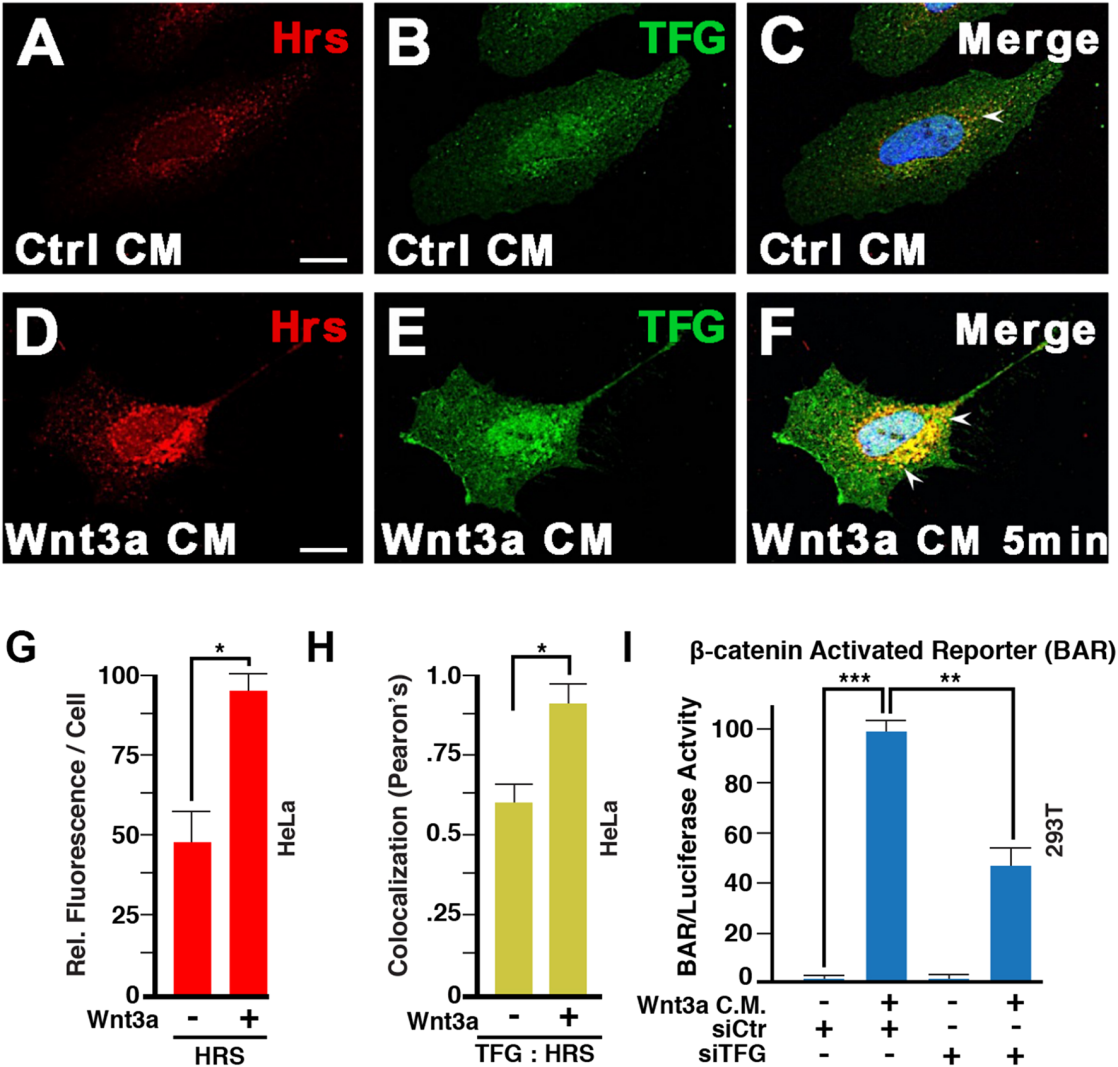
TFG co-localizes with the ESCRT-0 protein Hrs/Hgs and is required for Wnt signaling. (**A**–**F**) Immunostaining on HeLa cells for endogenous Hrs and TFG. Weak co-localization was observed in absence of Wnt signaling (arrow in panel **C**). However, 5 min of Wnt3a treatment was sufficient to strikingly increase the number of endocytic vesicles containing both Hrs and TFG (arrows). Scale bar 10 µm. (**G**) Wnt signaling (5 min) increased the number of Hrs positive vesicles quantified as fluorescence per cell; this supports the rapid formation of MVBs in Wnt signaling. (**H**) Wnt3a increased co-localization of TFG puncta with the Hrs MVB marker by Pearson’s correlation coefficient. (**I**) TFG knock-down by siRNA reduced Wnt signaling, as assessed by β-catenin Activated Reporter (BAR/Renilla) Luciferase assay in HEK293T cells.

Unexpectedly, one of the most highly enriched proteins upon 20 min of Wnt3a treatment was TFG (Fig. 2D), a protein containing multiple domains (Fig. S4A). N-terminal in-frame fusions of TFG to other genes are found in thyroid and lung cancers^26,39,40^. Notably, immunostaining of untransfected HeLa cells revealed endogenous TFG puncta that co-localized with Hrs (Fig. 3A–C), an ESCRT-0 protein that is required for Wnt signaling^11^. Hrs puncta, as well as their co-localization with TFG, were increased by Wnt3a after only 5 min of treatment (Fig. 3D–F, quantified in Fig. 3G,H). Co-localization of TFG and Hrs was confirmed by co-transfection of TFG-flag and Hrs-GFP constructs, with both proteins enriched in the periphery of endocytic vesicles (Fig. S4B–D). Co-immunostaining revealed close association between Lrp6-APEX2 and TFG even in the absence of Wnt (Fig. S4E–G). To investigate the function of TFG during Wnt signaling, we performed knock-down experiments in cultured cells. As shown in Fig. 3I, transfection of siRNA against TFG for two days decreased signaling by the Wnt/β-catenin-driven BAR luciferase reporter by over 50%, indicating that TFG is required for canonical Wnt signaling in cultured cells.

**Figure 4:**
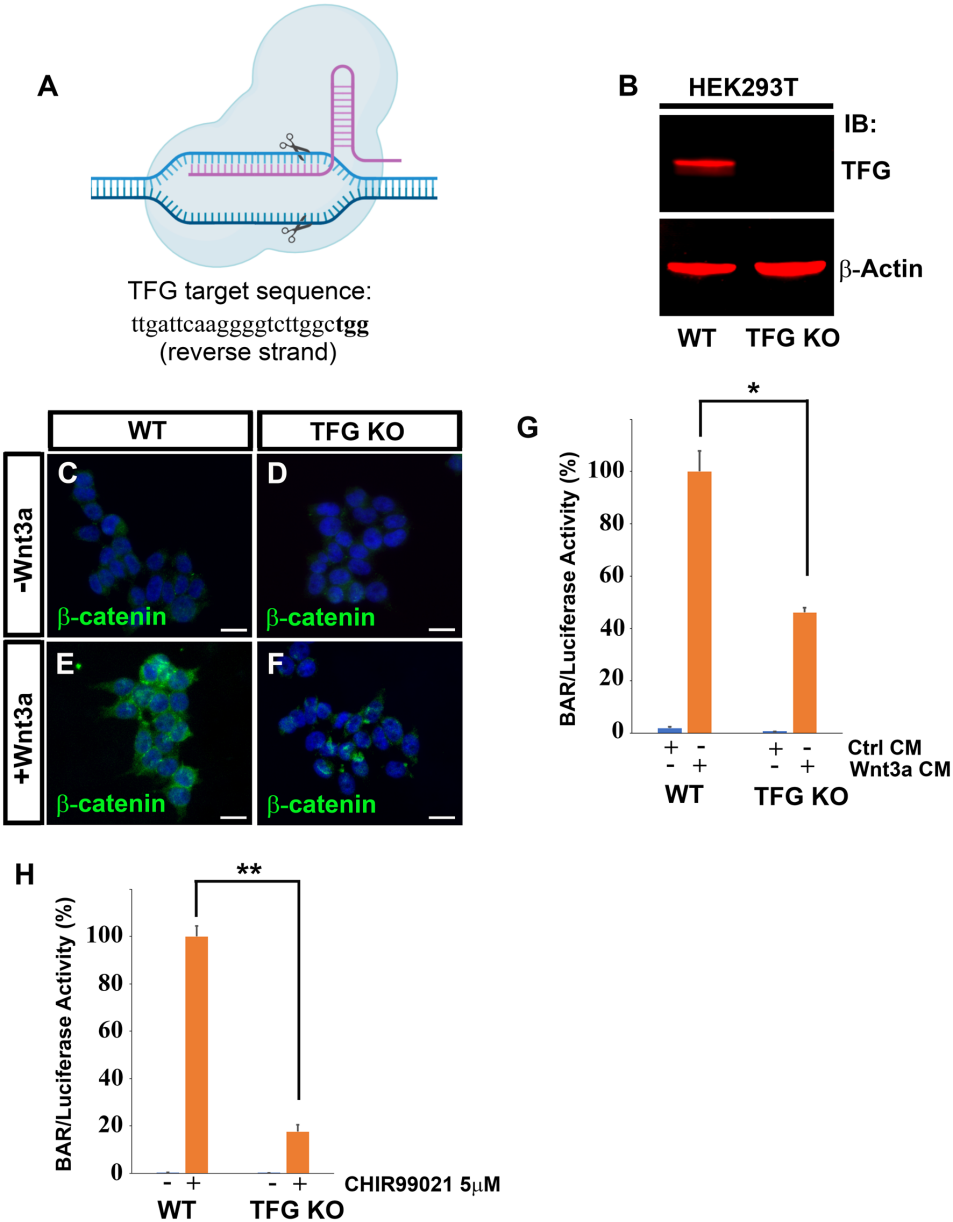
CRISPR-Cas9 mediated TFG knock-out inhibits Wnt-dependent β-catenin stabilization and Wnt-induced reporter activity. (**A**) Schematic diagram of Cas9 cleaving the TFG genomic DNA target sequence. The target sequence (which is on the reverse strand) is shown in 5′–3′ orientation (left–right); the PAM (protospacer adjacent motif) sequence is in bold. (**B**) Western blot of HEK293T cells confirming complete elimination of TFG protein in Cas9 knock-out cells. β-actin was used as loading control. (**C**–**F**) Immunostaining for β-catenin on WT or TFG KO cells. Cells were treated with control conditioned medium or with Wnt3a conditioned medium for 3 h before immunostaining. Note that TFG knock-out decreases Wnt3a-induced β-catenin accumulation; DAPI was used for nuclear counter-staining. Scale bars represent 20 µm. (**G**) Cas9-mediated TFG knock-out reduces response to Wnt3a by 50% in HEK293T BAR/Renilla β-catenin reporter cells. Cells were treated with Wnt3a or control medium for 16 h before luciferase analysis. (**H**) Luciferase assay of HEK293T BAR reporter cells treated with the GSK3 inhibitor CHIR99021 at 5 µM concentration for 16 h before being processed for luciferase assay. Note that TFG KO causes an 80% reduction of β-catenin luciferase activity in response to the GSK3 inhibition. Error bars represent standard deviation from triplicate experiments. Statistical significance was calculated with a paired 2-tailed t-Student test. *P < 0.05; **P < 0.01.

To further demonstrate the role of TFG in regulating Wnt signaling, we used the CRISPR/Cas9 (Clustered regularly interspaced short palindromic repeats/CRISPR-associated 9) gene editing technology^41–43^ to achieve full TFG knock-out. A guide RNA (sgRNA) targeting a sequence proximal to TFG start codon (Fig. 4A) was subcloned into a Cas9 expression vector and transfected into HEK293T cells harboring BAR Luciferase/Renilla reporters^15^. Western blot analyses confirmed complete depletion of TFG protein in a knock-out clonal cell line derived by limiting dilution (Fig. 4B). This knock-out line had two independent frameshift mutations caused by insertion/deletion (indel). Both mutations introduced an early termination codon, resulting in truncated TFG proteins of approximately 90 amino acids (Fig. S5). Next, we studied Wnt signaling in TFG knock-out cells. In wild-type (WT) cells, treatment with Wnt3a CM for 3 h induced a substantial increase in β-catenin immunostaining in WT cells (Fig. 4C,E), whereas TFG KO cells showed some increase in β-catenin upon Wnt3a treatment but at much reduced extent when compared to WT cells (Fig. 4*,* compare panels E and F). This suggested that TFG was required for maximal Wnt-induced β-catenin stabilization. Moreover, deletion of TFG reduced Wnt-driven BAR-luciferase reporter activity by over 50% compared to WT cells (Fig. 4G), in agreement with our previous observations with TFG siRNA. An even stronger reduction in β-catenin signaling (80%) was observed when we used CHIR99021, a specific GSK3 inhibitor, instead of Wnt3a in TFG mutant cells (Fig. 4H). While a requirement of TFG for GSK3 inhibition signaling was unexpected, it might be explained by our recent findings that CHIR99021 triggers massive macropinocytosis^38^; perhaps membrane trafficking, in which TFG is involved, is required for β-catenin stabilization. Taken together, the results suggest that the Lrp6 interacting protein TFG is indeed a novel component of the canonical Wnt signaling pathway.

### TFG is required for Wnt signaling in *Xenopus* embryos

To address the developmental role of TFG in vivo, we turned to *Xenopus* embryos as a model of choice. RT-PCR data showed that TFG was a maternal gene and its expression remained uniform until late stages of development (Fig. S4H). Unlike Chordin, a prototypical Spemann organizer gene^44^, TFG did not show dorso-ventral polarity (Fig. S4I). In situ hybridization showed that TFG was expressed in the animal pole of early cleavage embryos, and was later strongly expressed in the cement gland, as reported by others (Fig. S4J–N)^45^, in addition to staining in the notochord as shown here (Fig. S4O).

**Figure 5:**
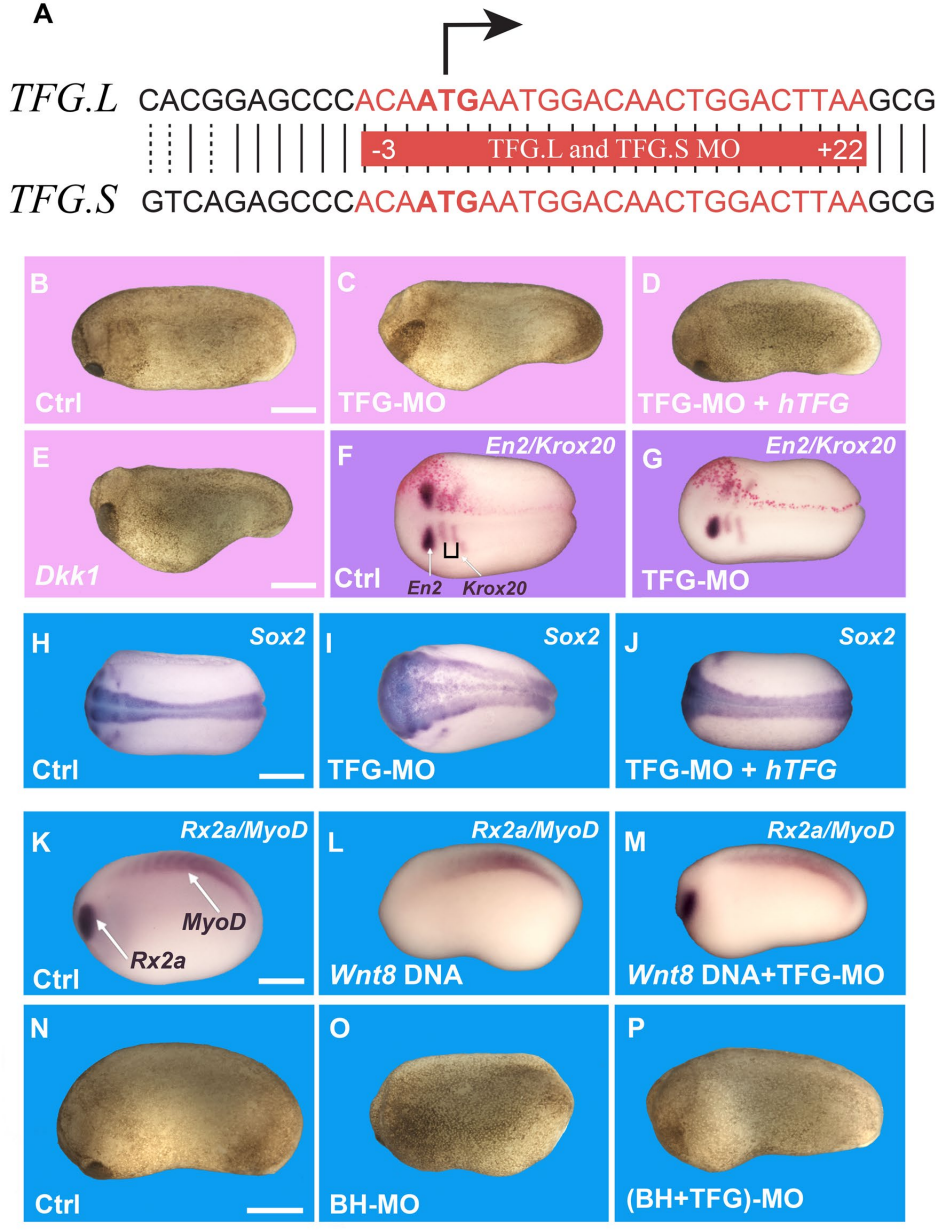
TFG regulates antero-posterior (A-P) patterning in the *Xenopus* embryo and is required for Wnt activity. (**A**) Diagram showing the TFG morpholino (MO) target sequence (in red), which is the same for both *Xenopus* L and S homeologs and span the ATG initiation codon. (**B**) Stage 24 embryos injected with control morpholino (Co-MO), showing normal development (92%, n = 127). (**C**) Embryos injected with 36 ng of TFG-MO showed severe enlargement of the head region (65%, n = 196). (**D**) Normal antero-posterior development was rescued by co-injecting 400 pg/embryo of human Flag-tagged *TFG* mRNA (77%, n = 52). (**E**) Embryos injected with 50 pg of mRNA encoding the Wnt antagonist Dkk1 showed head enlargement (100%, n = 35). (**F,G**) Embryos injected unilaterally with 18 ng of Co-MO or TFG-MO, together with 200 pg of nuclear *LacZ* mRNA, processed for in situ hybridization for *Engrailed-2* (*En2*) and *Krox20*. LacZ lineage tracing (in red) shows the injected side. TFG-MO reduces *En2* expression and shifts *Krox20* posteriorly in 65% of the embryos (n = 20), compared to control MO (n = 33). (**H,I**) In situ hybridization for the pan-neural marker *Sox2* at stage 20 showing enlargement of the neural plate following injection 4 times at the 2-cell stage with a total of 72 ng of TFG-MO per embryo (70%, n = 65), compared to the Ctrl-MO embryos (n = 35). (**J**) Co-injection with 800 pg of *hTFG* mRNA rescued the neural plate expansion (78%, n = 23). (**K**,**M**) Control embryos at stage 22 showed normal eye (*Rx2a*) and somite (*MyoD*) development (n = 18), while embryos injected with 32 pg of *xWnt8* DNA together with 72 ng of scrambled Ctrl-MO showed reduced or no *Rx2a* expression, but maintained *MyoD* expression (66%, n = 18). However, embryos injected with the same amount of *Wnt8* DNA together with 72 ng of TFG-MO showed rescue of *Rx2a* expression (84%, n = 32), indicating that TFG is required for xWnt8 signaling. (**N**–**P**) Embryos injected with Bighead morpholino (BH-MO) showed reduced head and cement gland development (71%, n = 21), compared to control embryos (n = 37), due to an increase in endogenous Wnt signaling (31). Co-injecting TFG-MO rescued normal head development in Bighead MO embryos (90%). Scale bars represent 500 µm.

To perform loss of function studies in vivo, we designed a morpholino antisense oligo^46^ which targeted both *Xenopus laevis* TFG homeologs (Fig. 5A) and efficiently blocked the translation of microinjected *Xenopus* flag-tagged *TFG* mRNA but not human *TFG* mRNA differing in the target sequence (Fig. S6A). Embryos microinjected with TFG morpholino displayed a strong anteriorization, with an enlargement of the cement gland and head (Fig. 5B,C, and Fig. S6B). Head enlargement was confirmed by in situ hybridization using the anterior marker *Otx2* (Fig. S6C,D). Co-injection of human Flag-tagged *TFG* mRNA completely rescued the anteriorizing effect, demonstrating that the phenotype of the TFG morpholino oligo was specific (Fig. 5D). Overexpression of human or *Xenopus* TFG mRNAs were without phenotypic effects on their own. However, human TFG lacking the MO target sequences was able to rescue the large head structures caused by TFG knockdown (Fig. 5D). The phenotype of TFG knock-down was very similar to that caused by the overexpression of known Wnt antagonists, such as Dkk1 (Fig. 5E), indicating that TFG is required for Wnt signaling in vivo. In line with this, TFG loss of function in one half of the embryo (marked by co-injection of nuclear *LacZ* mRNA) caused a marked decrease in the expression of the Wnt target gene *Engrailed-2* (Fig. 5F,G), while expression of the hindbrain marker *Krox20* was still present although shifted posteriorly. The pan-neural marker *Sox2* was expanded by TFG morpholino, an effect also observed upon Wnt inhibition^47^, and was rescued by co-injection with human *TFG* mRNA (Fig. 5H–J).

Next, we injected xWnt8 DNA into *Xenopus* embryos that, as expected^48^, caused the inhibition of anterior structures such as eyes (Fig. 5K,L) and cement gland (Fig. S6E,F). However, injection of TFG-MO together with Wnt8 DNA rescued eye and cement gland development (Fig. 5M and Fig. S6E–G), indicating that TFG is required for Wnt activity in vivo. To show that TFG-MO is required for endogenous late Wnt signaling we used knock-down of the Wnt antagonist Bighead, which is known to result in an increase of Wnt activity^31^. We found that while Bighead-MO caused loss of anterior structures in *Xenopus* embryos, this effect could be rescued by co-injection of TFG-MO (Fig. 5N–P), indicating that TFG functions downstream of Lrp6. Taken together, the results indicate that the Lrp6-APEX2 interacting protein TFG is required for Wnt/β-catenin signaling in *Xenopus* development.

## Discussion

**Figure 6:**
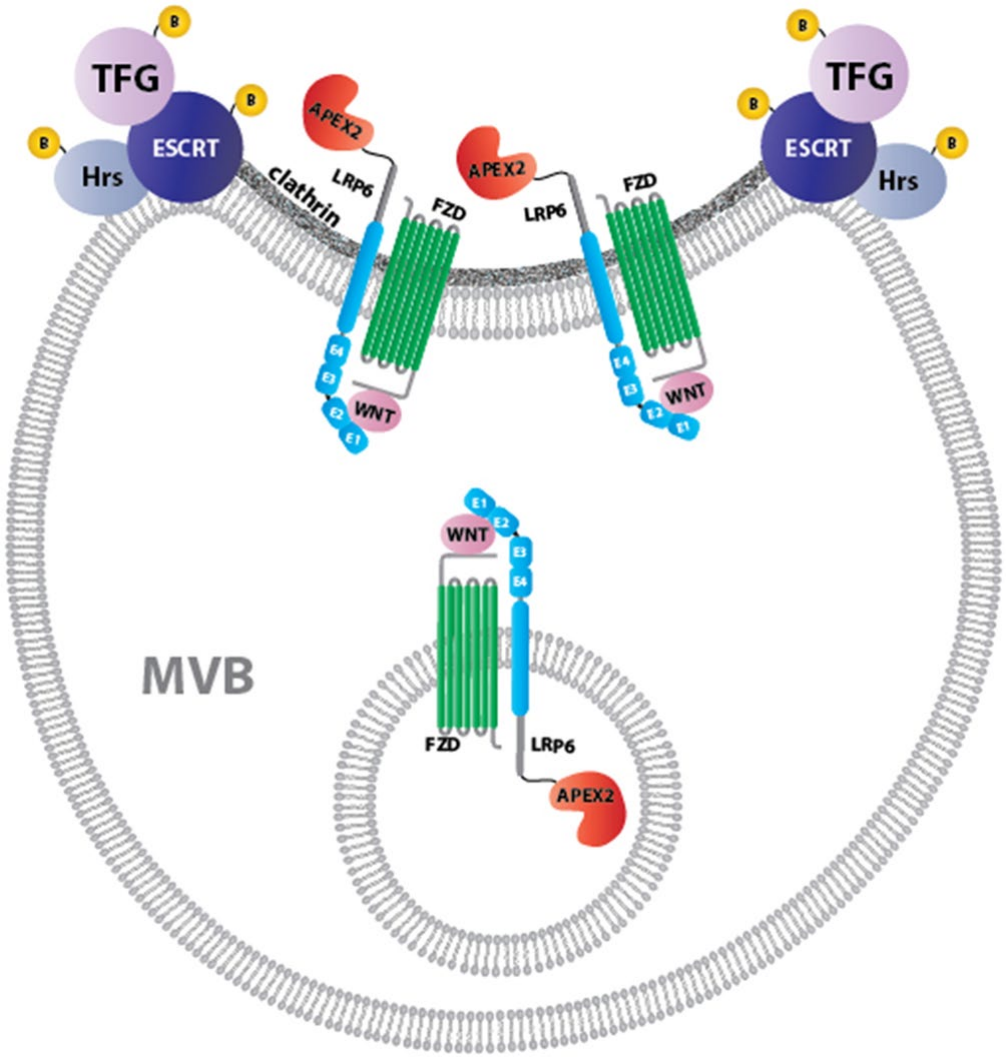
Model of how Lrp6-APEX2 may interact with the ESCRT machinery and TFG in multivesicular body formation during Wnt-induced endocytosis. The endocytosed Wnt receptor complex on the surface of multivesicular endosomes becomes clustered in clathrin-containing invaginations that give rise to the intraluminal vesicles (ILVs) of late endosomes. MVBs are the obligatory pathway through which plasma membrane proteins reach the lysosome. We found that most ESCRT GO components are brought into proximity of Lrp6-APEX2 5 min after treatment with Wnt3a conditioned medium. The top interactor with Lrp6-APEX2 after 20 min of Wnt3a was TFG, which we show here is required for Wnt signaling. TFG protein co-localizes with the ESCRT-0 protein Hrs/Hgs after Wnt stimulation. We did not detect interactions with intraluminal vesicle components such as CD63; a possible explanation is that the acidic medium or lysosomal proteases might interfere with APEX2 activity.

In the present study, we used biotin-dependent proximity labeling to study the early molecular interactions following Wnt/β-catenin activation. Proteomic analyses generated a searchable list of Lrp6 interactors that is provided in Supplementary Table S1, and can be mined by the research community to identify additional Wnt regulators. Importantly, our data showed that ESCRT proteins such as Hrs/Hgs, Stam1/2, CHMP4, Vps28, Vps37 and Tsg101 were recruited close to the Lrp6 Wnt receptor soon after Wnt stimulation (5 min) and were still significantly enriched 20 min later. In agreement with this, our immunostaining on cultured cells showed that 5 min of Wnt3a treatment was sufficient to significantly increase the number of Hrs-containing endocytic vesicles. Thus, the results presented here strongly support the view that multivesicular endosome formation is a key downstream step in Wnt signaling^11,12^, as indicated in the model shown in Fig. 6. Our proteomic data also indicate a dynamic enrichment of different proteins to Lrp6, over time, as one would expect from a signaling cascade. According to our data, the earliest event would be the recruitment of endosome-associated protein, triggering the endocytosis of the activated Wnt receptor. This is immediately followed by the recruitment of Wnt signalosome proteins (including Axin, Dishevelled etc.) whose enrichment begins already at 5 min, but is maximized at later time point (20 min in our analysis). Perhaps the assembly of the Wnt signalosome is stabilized upon endocytosis of the receptor complex. Of note, APC was enriched at 5 min, but strongly down-regulated at 20 min. APC has been shown to inhibit clathrin-mediated endocytosis of Lrp6, a process that prevent inadvertent activation of Wnt pathway^29^. It is possible that, upon Wnt stimulation, APC is removed from Lrp6, which is then endocytosed into MVBs together with other signalosome components.

The connection between Wnt and endocytosis is well known^49,50^, and several factors have been found to modulate Wnt through endosomal mechanisms. For example, the protein arginine methyltransferase 1 (PRMT1), which interacts with Lrp6^51^, was shown to be indispensable for the sequestration of GSK3 into MVBs by microautophagy^15,35^ in order to sustain canonical Wnt signaling. In recent work, a proteomic proximity approach similar to the one reported here for Lrp6-APEX2, made use of a Fzd9b-APEX2 fusion to discover that Epidermal Growth Factor Receptor (EGFR)-mediated phosphorylation of Fzd9b was required for receptor internalization and signal transduction in response to Wnt9a^52^. In that study, the proteomic data showed that endosome-associated Rabs and the Clathrin-endocytic pathway interacted with Fzd9b upon Wnt9a signaling, supporting the view that the endosomal machinery is an early component of the Wnt/β-catenin pathway. Importantly, in this study we found that Kras, which is a major regulator of macropinocytosis^53^, interacts with Lrp6-APEX2 after Wnt treatment. A central role for endocytosis in Wnt signaling is consistent with the observations reported here, which revealed striking interactions with the ESCRT machinery that generates MVBs after Wnt treatment (Fig. 2B). In addition, Wnt3a treatment induced interactions between Lrp6 and components of the actin cytoskeletal machinery, which was particularly prominent at early (5 min) and later time points (1 h). This finding was expected, as endocytosis requires remodelling of the cytoskeleton and plasma membrane. While the exact molecular links between Wnt, endosomes and actin cytoskeleton are still unclear, the data presented here support our recent findings that Wnt is essential for macropinocytosis, an actin-driven endocytic process with important consequences on cell metabolism that requires Gsk3 inhibition^16,37,38^.

The best validation of the proximity biotinylation approach was the identification of an unexpected player in the Wnt pathway. Among the most enriched Lrp6 interactors we found TFG, a protein that was first identified in papillary thyroid carcinomas as a result of an in-frame oncogenic rearrangement between its N-terminal region and the tyrosine kinase domain of Neurotrophic tyrosine kinase receptor type 1 (NTRK1, also designated as Trk)^39^. The ability to self-oligomerize, conferred by the TFG N-terminal coiled-coil domain, leads to constitutive activation of the tyrosine kinase domain responsible for the transforming activity of this chimeric oncogenic protein^40^. Similarly, translocations between the TFG and anaplastic lymphoma kinase (ALK) genes result in a fusion oncoprotein in some lung cancers^54^. The physiological function of wild type TFG has been previously investigated. Studies in *C. elegans* and human cultured cells reported that TFG localizes to the ER exit sites adjacent to the ER-Golgi intermediate compartment known as the ERGIC compartment, a cluster of membranes that secretory cargos traverse on their route to the Golgi apparatus^55^. TFG hexamers were shown to co-localize and interact with the coat protein complex II (COPII) components Sec16 and Sec23, promoting the trafficking and secretion of cargo proteins, including synaptobrevin^55,56^. The importance of TFG in membrane traffic is also highlighted by the presence of several pathogenic mutations that impair TFG activity in ER function and are linked to different neurological disorders, including a hereditary motor and sensory neuropathy (HMSN-P)^57^.

Our findings revealed an additional function for TFG, a protein that localized to the same endosomal vesicles as Hrs. Wnt3a treatment, which increases the number of late endosomes/MVBs^15,16^, stimulated the co-localization of TFG and HRS. Interactions between TFG, Hrs and other components of the ESCRT machinery such as Tsg101 have been reported on BioGRID (https://thebiogrid.org/), a repository for protein–protein interactions that curates data derived from high-throughput datasets. Moreover, β-catenin reporter assays in cell culture showed that TFG is required for Wnt signaling, as confirmed both by siRNA mediated knock-down and CRISPR/Cas9 knock-out of TFG. Our Cas9 genome editing experiments generated a cell line expressing only the first 90 amino acids of TFG. The resulting short protein lacks the coiled-coil domain, which spans from amino acids 97 to 124^39^. This detail is of importance, as the coiled coil domain is involved in TFG oligomerization and is required for its function and transforming activity^40^, suggesting that our truncated TFG acts potentially as a null mutant that resulted in a 50% inhibition of canonical Wnt signaling. We do not know why the effect of TFG loss of function is only partial on stimulation by Wnt ligands, but we speculate that structurally related proteins may partially compensate for TFG absence.

The requirement for TFG during Wnt signaling is strongly supported by experiments in *Xenopus laevis* embryos. In vivo loss of function experiments using a TFG morpholino showed that TFG was required for correct antero-posterior (A-P) patterning and head development in frog embryos. In *Xenopus*, A-P patterning is regulated by an activity gradient of Wnt signaling which is lower in the rostral (head) region of the embryo^58^. Numerous secreted Wnt inhibitors, including Dkk1^30^, Frzb^59^ and Bighead^31^ are required to maintain low levels of Wnt and allow head development. Embryos injected with TFG morpholino developed larger heads and cement glands, mimicking the effects of Dkk1 overexpression and pointing to a reduction of the endogenous Wnt signal. In agreement with this view, TFG knock-down also abolished the posteriorizing activity exerted by Wnt8 DNA overexpression or Bighead knockdown, showing in vivo that Wnt signaling requires TFG. At present, the molecular mechanism by which TFG regulates the Wnt pathway is unknown, but the co-localization with the ESCRT-0 component Hrs points to a role in the MVB compartment (Fig. 6). Future work will determine whether other Wnt-induced interactions of Lrp6, provided here as an open resource to the community in Supplementary Table S1, also participate in canonical Wnt signaling. We also conclude that Lrp6 endocytosis plays an important role in canonical Wnt signal transduction.

## Methods

### Cloning and plasmids

pCS2 was used as a backbone for cloning recombinant DNA. First, the basic pCS2 plasmid was made compatible with the Gateway recombination system, by cloning a ccdb/chloramphenicol cassette (Invitrogen, 11828029) into the pCS2 StuI restriction site. Flag-tagged cytosolic APEX2 (Addgene, 49386) was then amplified by polymerase chain reaction (PCR) and sub-cloned in frame with the 3′ of the Gateway cassette, into the pCS2 XhoI/XbaI sites. VSV-G-tagged human Lrp6 was obtained from Addgene (27282), amplified by PCR using primers containing Gateway sequences, and sub-cloned through a Gateway reaction following manufacturer’s instructions. Finally, an IRES sequence followed by a GFP-tagged Puromycin N-Acetyltransferase (PAC) gene, obtained from Addgene plasmid 45567, was amplified by PCR and sub-cloned into XbaI/SnaBI sites, downstream of the Lrp6-APEX2 open reading frame (ORF), to allow for selection of stable transfectants with the antibiotic puromycin. A human TFG clone was obtained from Dharmacon, sequence verified, and Gateway-cloned into a pCS2 vector containing a 3xFlag epitope tag at the C-terminus. *Xenopus laevis* TFG was PCR-amplified from cDNA obtained from frog eggs, Gateway-cloned into pCS2-3xFlag and pCS2-Myc-Streptact. All cloned sequences were verified by DNA Sanger sequencing (Genewiz). Primer sequences are shown on Table S1. The plasmids used in this study will be made available on the Addgene plasmid repository. All animal experiments procedures were approved by the UCLA institutional Animal Research Committee.

### Stable line transfection

The pCS2 Lrp6-APEX2-IRES-GFP-PAC plasmid was transfected into Human Embryonic Kidney 293 T (HEK293T) cells using Lipofectamine2000 (Thermo Fisher Sci.). 48 h after transfection puromycin was added to the culture medium, at a final concentration of 4 ug/ml. Stable transfected cells were continuously expanded and selected with antibiotic for at least two weeks, after which monoclonal lines were derived by limiting dilution. Two independent clones were obtained, which maintained stable and uniform expression of the Lrp6-APEX2 and GFP-PAC chimeras after many cell passages.

### Mass spectrometry analyses

Affinity-purified biotinylated protein samples were reduced and alkylated using 5 mM Tris (2-carboxyethyl) phosphine and 10 mM iodoacetamide, respectively, and then proteolyzed by the sequential addition of trypsin and lys-C proteases at 37 °C as described^33,34^. The digestion reactions were stopped by the addition of 5% formic acid, desalted using Pierce C18 tips (Thermo Fisher Sci.), and then dried and resuspended in 5% formic acid. Peptide digests were fractionated online using a 25 cm long, 75 µm inner diameter fused silica capillary packed in-house with bulk C18 reversed phase resin (1.9 µm, 100A pores, Dr. Maisch GmbH). A 140-min water-acetonitrile gradient was delivered to a maximum of 80% buffer B using a Dionex Ultimate 3000 UHPLC system (Thermo Fisher Sci.) at a flow rate of 300 nl/minute (Buffer A: water with 3% DMSO and 0.1% formic acid and Buffer B: acetonitrile with 3% DMSO and 0.1% formic acid). Peptides were ionized using a distal 2.2 kV spray voltage and a capillary temperature of 275 °C and analyzed by tandem mass spectrometry (MS/MS) using an Orbitrap Fusion Lumos mass spectrometer (Thermo Fisher Sci.). Data was acquired by a Data-Dependent Acquisition (DDA) method comprised of a full MS1 scan at 120,000 resolution followed by sequential MS2 scans (Resolution = 15,000) to utilize the remainder of the 3 s cycle time. Data analysis was performed using two discrete bioinformatic pipelines. The first analysis used the Integrated Proteomics pipeline 2 (Integrated Proteomics Applications, San Diego, CA) to generate peptide and protein lists that were quantified using spectra counting. In this case, MS2 spectra were searched using the ProLuCID algorithm against the EMBL Human reference proteome (UP000005640 9606) followed by filtering of peptide-to-spectrum matches (PSMs) by DTASelect using a decoy database-estimated false discovery rate of < 1%. In the second approach, an in-house Galaxy-based pipeline was used to provide label-free MS1 scan intensity-based quantification for the samples. MS2 spectra were searched against the EMBL Human reference proteome (UP000005640 9606) using the MSGF + search engine^60^. False detection rates for evaluating the peptide-spectrum-match (PSMs) were determined using Percolator while protein identification confidence was estimated via the stand-alone implementation of FIDO such that analytes had respective score cut-off q-values at or below 0.01 at both PSM and protein level^61–63^. The Integrated peak areas of extracted ion chromatograms across multiple runs were calculated for each peptide using the Skyline software platform^64^. In order to calculate the fold change and determine the protein abundance changes, the MSStats R-package was used to normalize across runs using quantile normalization, summarize peptide-level intensities into a protein-level abundance, and perform statistical testing to compare protein abundance across conditions^65^. The raw dataset generated by mass spectrometry has been deposited in the online mass spectrometry interactive virtual environment (MassIVE) repository resource, with accession number MSV000084335. Additional methods, including proximity labeling, embryo and cell culture procedures are available in Supplementary Information.

## Supplementary information

Supplementary Information 1.

Supplementary Video 1.

Supplementary Information 2.
