# Supplementary material for: Wnt-inducible Lrp6-APEX2 interacting proteins identify ESCRT machinery and Trk-fused gene as components of the Wnt signaling pathway
Source: Sci Rep. 2020 Dec 9;10:21555. doi: 10.1038/s41598-020-78019-5 (PMC7726150; doi:10.1038/s41598-020-78019-5)
Supplement: Supplementary file 3 — Supplementary Information 2. [file 41598_2020_78019_MOESM3_ESM.pdf]

Supplementary Information for

Wnt-inducible Lrp6-APEX2 Interacting Proteins identify ESCRT Machinery and Trk-Fused Gene as Components of the Wnt Signaling Pathway

Gabriele Colozza, Yasaman Jami-Alahmadi, Alyssa Dsouza, Nydia Tejeda-Muñoz, Lauren V. Albrecht, Eric Sosa, James A. Wohlschlegel and Edward M. De Robertis

Corresponding authors: Gabriele Colozza; Edward M. De Robertis

Email: coloz80@gmail.com ; ederobertis@mednet.ucla.edu

This PDF file includes:

Figures S1-S5 and Legends

Tables S1, S2, S3

Captions for Movie S1

Supplemental Methods

Supplemental References

Other supplementary materials for this manuscript include the following:

Supplementary Movie S1

Supplementary Table S1

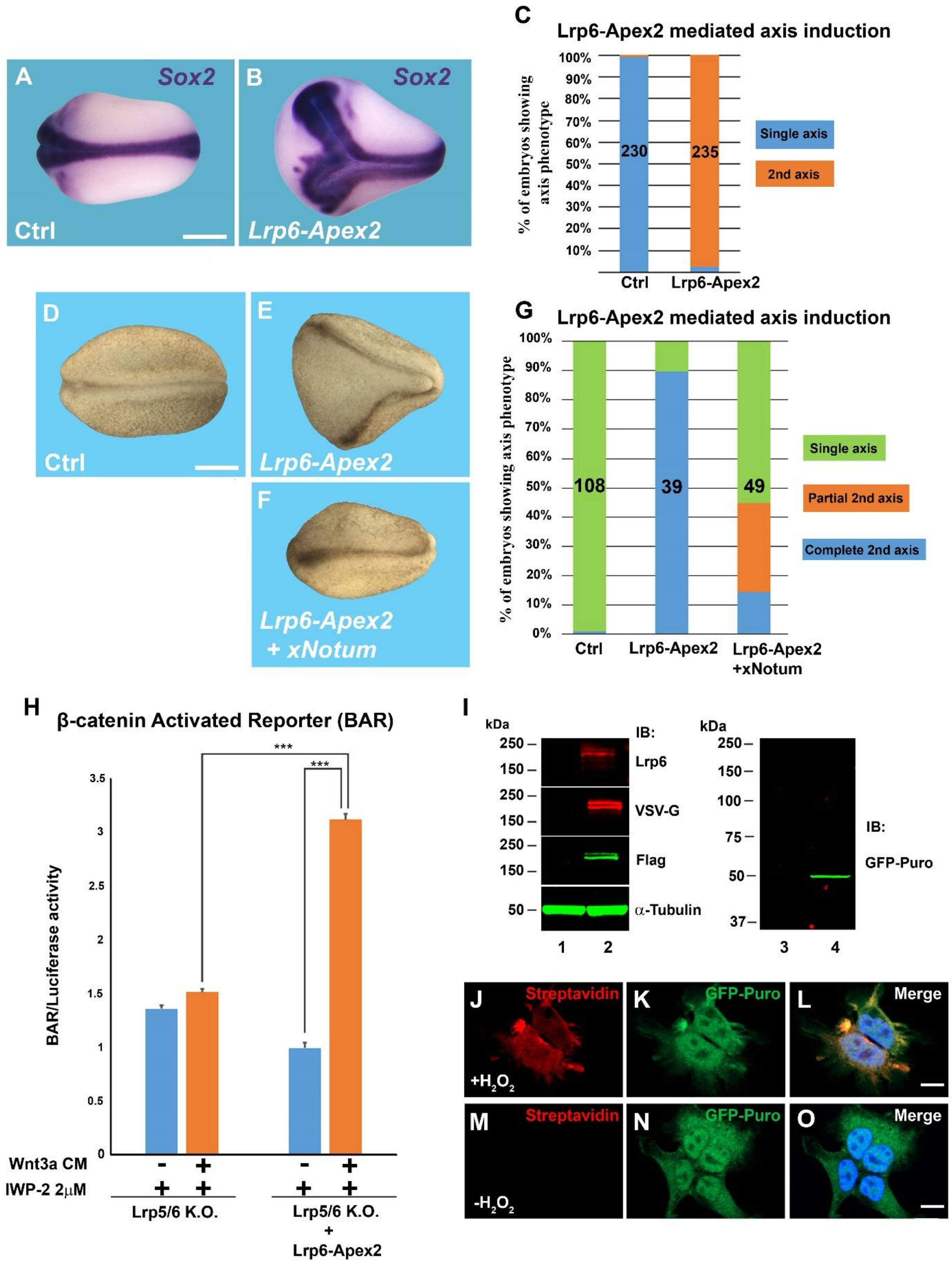

Fig. S1. ***Xenopus*** **embryo and cell-based assays demonstrate Lrp6-APEX2 transduces Wnt signaling.** (**A**, **B**) Microinjection of 1 ng of *Lrp6-APEX2* mRNA into one ventral blastomere of

Xenopus embryos at the 4-cell stage induced formation of a secondary axis, visualized through *in situ* hybridization for the neural marker *Sox2* at stage 20. Scale bar corresponds to 500 µm. (**C**) Quantification of the experiment shown in panels **A** and **B**. Lrp6-APEX2 induced double axes with a penetrance of almost 100%. The number of embryos used for these experiments are shown inside the columns. (**D**-**F**) Injection of 1 ng of *Lrp6-APEX2* mRNA into a single ventral blastomere of *Xenopus* embryos at the 4-cell stage induced secondary axes, but this required endogenous Wnt ligands as co-injection of 300 pg of mRNA encoding the Wnt de-acylase *Notum* strongly inhibited secondary axis formation. Embryos were fixed and scored at stage 20. Scale bar corresponds to 500 µm. (**G**) Quantification of the experiment shown in panels **D**-**F**. Lrp6-APEX2 induced double axes with a penetrance of 90%, and co-injection with the Wnt inhibitor *Notum* strongly inhibited this activity. (**H**) β-catenin-Activated reporter (BAR)-Luciferase assay showing that transient expression of Lrp6-APEX2 was able to rescue response to Wnt3a conditioned medium in Lrp5/6 double knock-out cells [1]. Double knock-out cells were preincubated overnight with 2 µM (final concentration) of the Porcupine inhibitor IWP-2 to prevent activation by endogenous Wnt signaling in the rescued cells. (**I**) Western Blot of whole cell lysates derived from parental HEK293T cells (lanes 1 and 3) or Lrp6-APEX2 293T clonal cells (lanes 2 and 4). Robust Lrp6 expression was detected with different anti-Lrp6 antibodies (Lrp6, VSV-G and Flag) in this permanent cell line. Note the presence of the two typical bands, with the lower one corresponding to the immature form of Lrp6, α-tubulin was used as a loading control. Lane 4 shows correct expression of a GFP-tagged selectable marker, puromycin N-acetyl-transferase, whose expression is driven by an IRES placed downstream of Lrp6-APEX2. (**J**-**O**) APEX2 peroxidase activity in Lrp6-APEX2 293T cells could be visualized by fluorescent Streptavidin, which binds to proteins biotinylated by APEX2. Note that Streptavidin fluorescence was observed only in presence of H_2O_2 (and biotin-phenol), which is the substrate for peroxidase activity. GFP-puromycin was used as counter-staining, and DAPI nuclear staining is shown in the Merge panels. Scale bars represent 10 µm.

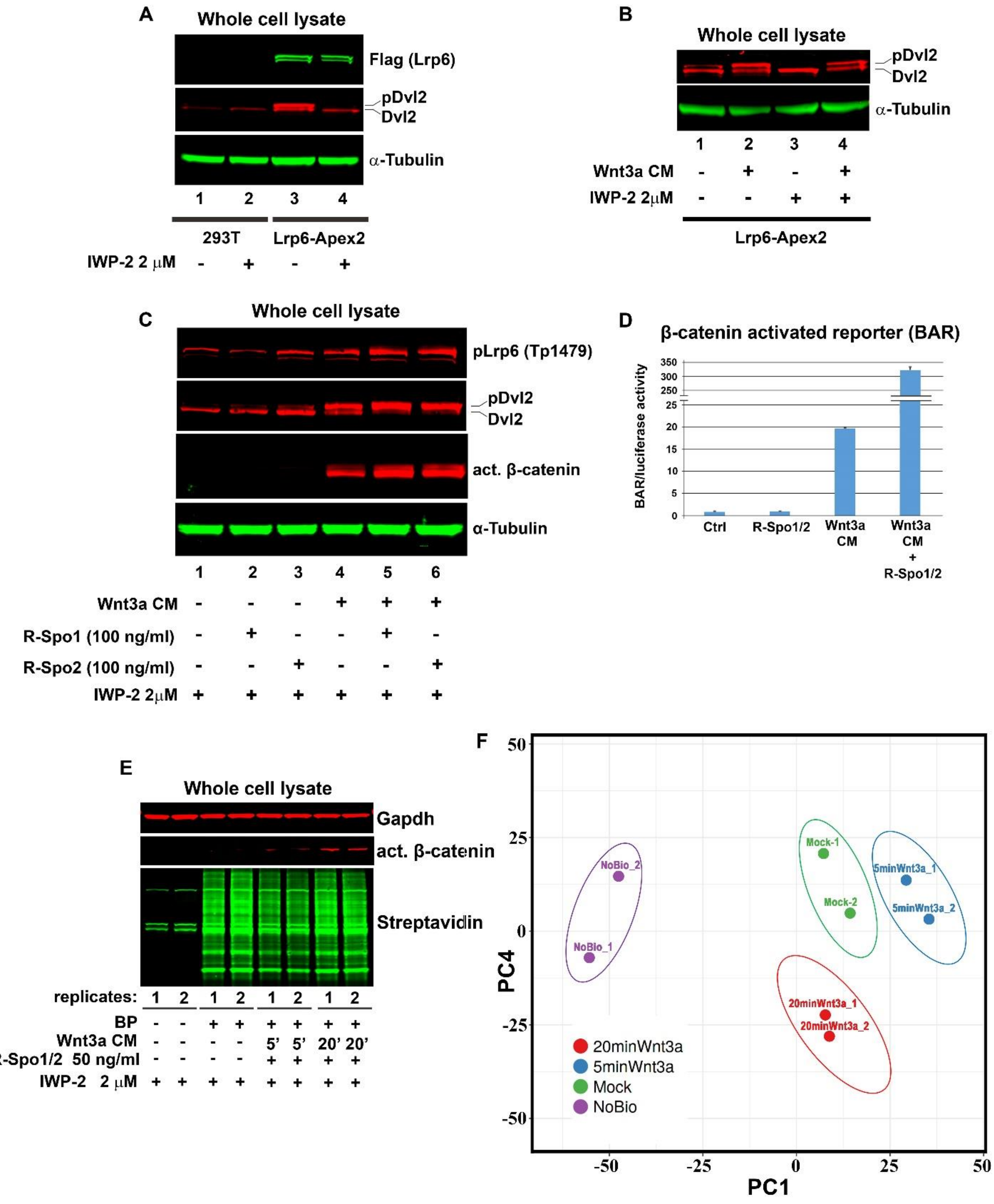

Fig. S2. Lrp6-APEX2 cells respond to exogenous Wnt signals in the presence of IWP-2, and biotinylation of key Wnt pathway proteins after Wnt addition. (**A**) Western blot of lysates from control HEK293T and Lrp6-APEX2 cells. Lrp6-APEX2 cells show high levels of

phosphorylated Dvl2 (pDvl2), a hallmark of activated Wnt signaling (lane 3), even in absence of exogenous Wnt. However, overnight incubation with 2 µM IWP-2, an inhibitor of the Wnt-palmitoyltransferase Porcupine, prevented Dvl2 phosphorylation (compare lanes 3 and 4). pDvl2 was not detected in 293T control cells (lanes 1 and 2) even in the absence of IWP-2 incubation, indicating that permanent transfection with Lrp6-APEX2 sensitizes cells to low levels of endogenous Wnt signals; α-tubulin was used as a loading control. (**B**) Western blot of Lrp6-APEX2 293T cell lysates. In absence of IWP-2, pDvl2 was detected regardless of Wnt3a conditioned medium (CM) treatments (lanes 1 and 2). However, Lrp6-APEX2 cells incubated overnight with IWP-2 showed Dvl2 phosphorylation only in the presence of Wnt3a CM (lanes 3 and 4); α-tubulin was used as a loading control. (**C**) Western blot of Lrp6-APEX2 cell lysates showing that cells responded robustly to Wnt3a CM and that this was further potentiated by R-spondins 1 and 2, even in the presence of Porcupine inhibitor IWP-2. Wnt3a CM induced robust Dvl2 phosphorylation, stabilization of non-phosphorylated (active) β-catenin, and Lrp6 phosphorylation at Threonine residue 1479 (compare lanes 1 and 4) after 3 hours of Wnt3a treatment. Wnt pathway activation was further increased when R-Spondin 1 and 2 recombinant proteins [2] were added to Wnt3a CM (lanes 5 and 6). R-Spo proteins alone had little effect on Wnt activation (because of the presence of IWP-2); α-tubulin was used as a loading control. (**D**) Wnt3a CM activity was tested on a permanently transfected HEK293T β-catenin activated reporter (BAR) cell line. Wnt3a CM induced a 20-fold increase in luciferase activity, while combination of Wnt3a CM and R-Spo1/2 proteins (50 ng/ml each) induced a 200-fold increase. This was adopted as the fortified Wnt3a CM formulation was used for proteomic experiments. (**E**) Proximity biotin-labelling of Lrp6-APEX2 cell in aliquots of the same large-scale cultures used in the proteomic experiments after 5 min or 20 min of treatment with Wnt3a CM containing recombinant R-Spo1/2 (50 ng/ml each). Western blot using Streptavidin-IRDye 800 was performed to confirm that biotinylation occurred (note that the biotin "ladder" was observed only in presence of Biotin-phenol, confirming specificity). The three bands seen in lanes 1 and 2 represent endogenous biotin-containing carboxylases. Non-phosphorylated Ser33/37/Thr41 β-catenin, which represents its active form (act. β-catenin) [3-5], confirmed Wnt pathway activation; Gapdh was used as a loading control. Experiments were conducted on biological duplicates, which were then processed for mass spectrometry. (**F**) Principal Component Analysis (PCA) to analyze the dimensionality and trends exhibited by the enrichment profiles of the different proteomic experiments. The samples included biological duplicates of 4 different cell treatments: no-biotinylation control (NoBio), treatment with control CM (Mock) and treatment with Wnt3a CM for 5 minutes (5minWnt3a) or 20 minutes (20minWnt3a). Each axis represents a principal component (PC1 and PC4). These PCA analyses confirmed reproducibility of experiments by clustering duplicate conditions. Each dot represents a sample and each color represents the type of treatment.

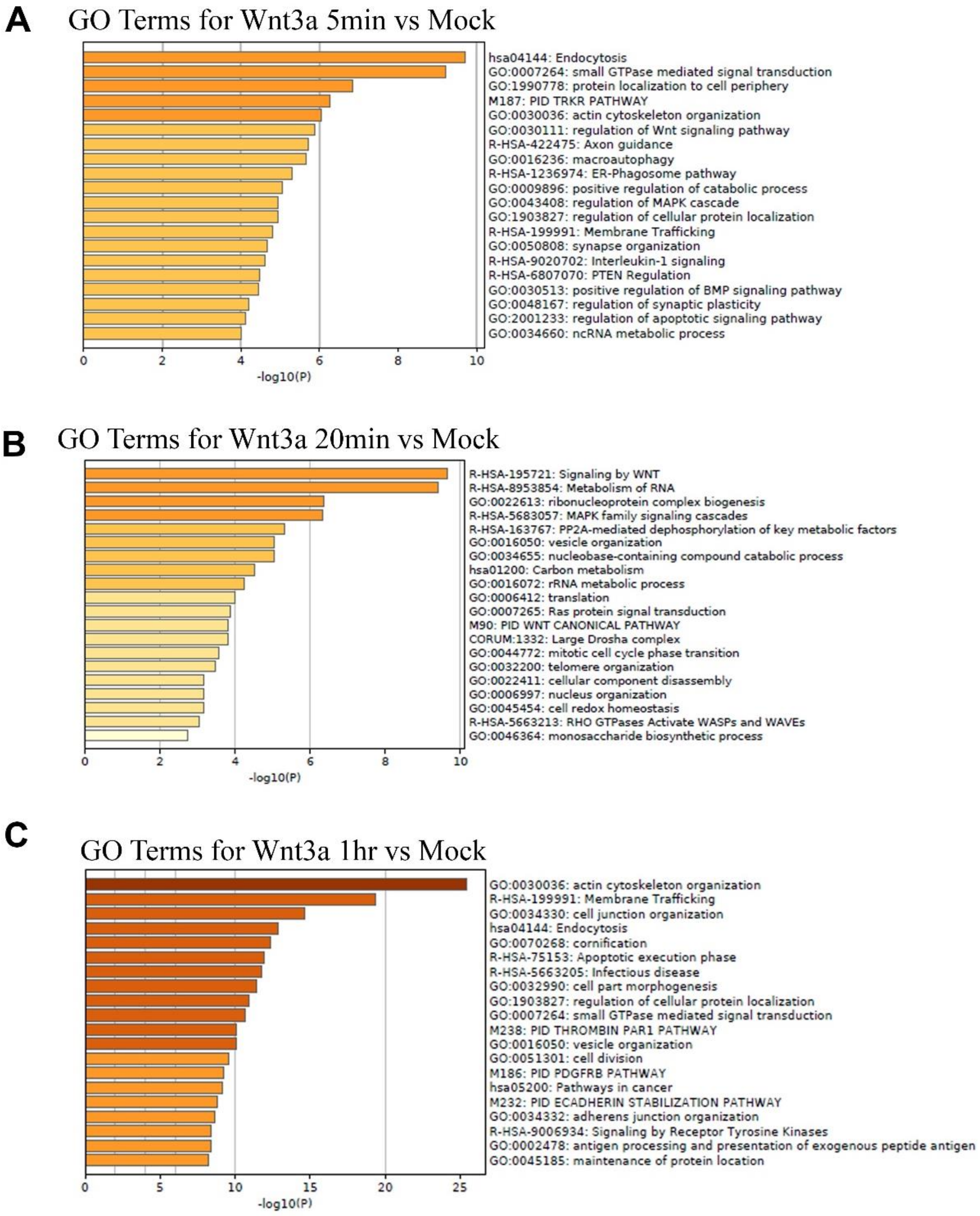

Fig. S3. Gene Ontology analyses of biotinylated Wnt-induced Lrp6-APEX2 target proteins at three timepoints. Biotinylated proteins from Wnt samples were ranked for enrichment against the control Mock samples (cells treated with control medium). The top hits (150-300 top proteins) were then analyzed with the Metascape online software [6], available at metascape.org/gp/index.html#/main/step1. (**A**) Analysis of the top 150 biotinylated Lrp6 interactors after 5 minutes of Wnt3a treatment. (**B**) Analysis of 130 biotinylated Lrp6 interactors after 20 minutes of Wnt3a treatment. (**C**) Analysis of 300 top biotinylated Lrp6 interactors after 1

hour of Wnt3a treatment. Bars represent the enriched terms across input protein lists, colored by p-values.

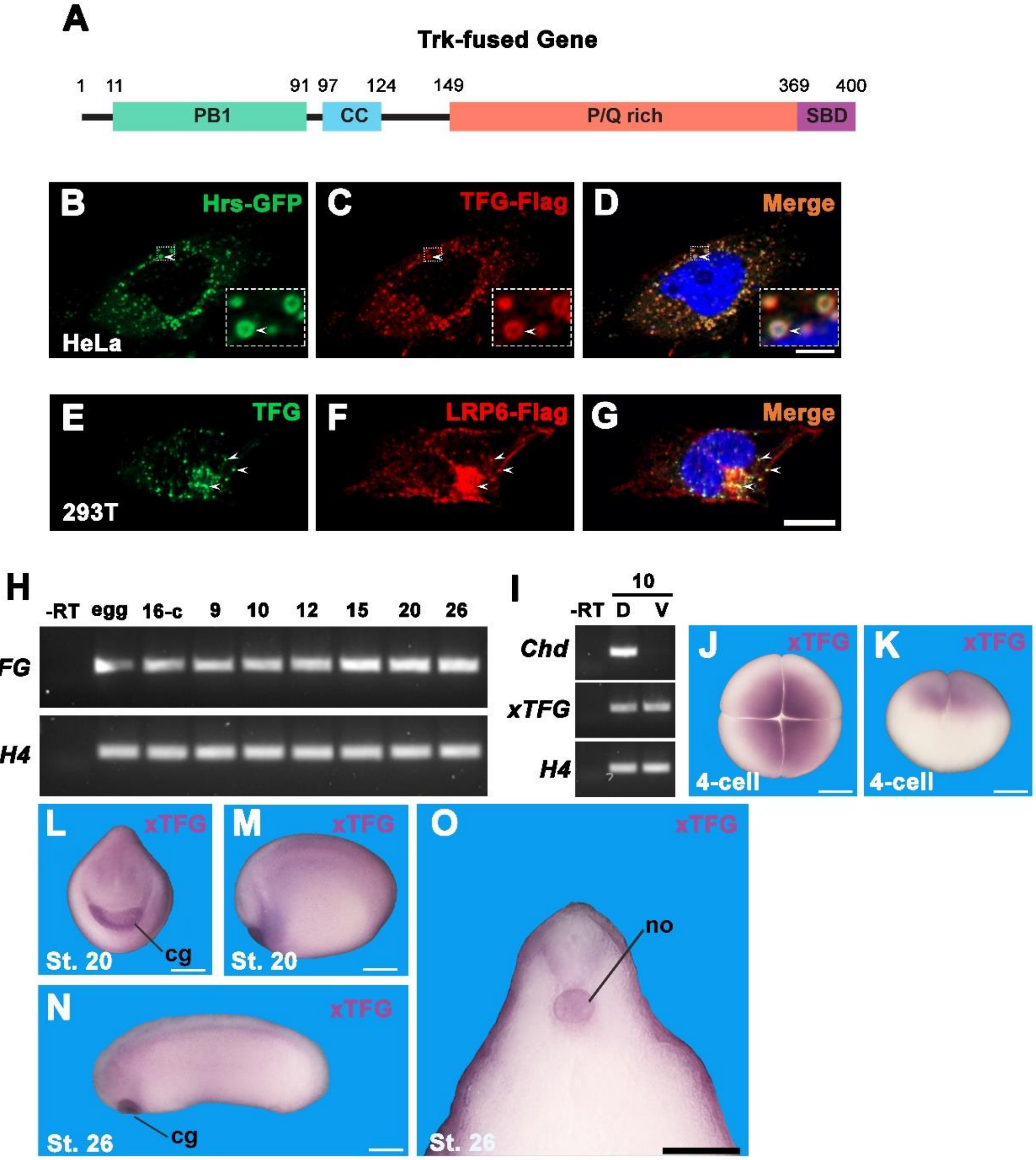

Fig. S4. TFG colocalizes with Hrs and Lrp6 and is expressed in developing *Xenopus laevis* embryos. (**A**) Diagram highlighting human TFG protein structure and its structural domains [7]. PB1, also called Phox and Bem1p domain, is possibly involved in heterodimer formation and is required for oncogenic activity of TFG fusion products; CC is a coiled-coil domain involved in oligomerization and also required for the transforming activity of oncogenic TFG fusions; P/Q rich is a Proline/Glutamine rich domain, a disordered region required for localization of TFG at the endoplasmic reticulum (ER)-Golgi intermediate compartment (ERGIC). SBD is the Sec23-binding domain, required for interaction with Sec23 and disassembly of COPII vesicles. Numbers indicate amino acid position. (**B**-**D**) Immunofluorescence on HeLa cells transfected with plasmid DNAs encoding mouse Hrs-GFP and human TFG-Flag. Note the strong colocalization between the two proteins. Inset shows overlay at the periphery of endocytic vesicles. These results strongly support

the specificity of endogenous HRS and TFG immunostainings shown in Fig. 3 of the main text. DAPI nuclear staining (in blue) is shown in Merge panel; scale bar represents 10 µm. (**E**-**G**) Immunofluorescence of Lrp6-APEX2 293T cells showing partial endogenous partial overlap with endogenous TFG (arrowheads), indicating co-localization between the two proteins and corroborating the results from proteomic analysis. DAPI nuclear staining (in blue) is shown in Merge panel; scale bar represents 10 µm. (**H**) RT-PCR analysis on cDNA obtained from *Xenopus* embryos at different developmental stages. *xTFG* mRNA was present as a maternal factor (in eggs and 16-cell stage embryos) and continued to be expressed at later stages. *Histone 4* (*H4*) was used as a loading control. -RT was used as a negative control. (**I**) RT-PCR on dorsal (D) and ventral (V) fragments dissected from stage 10 *Xenopus* gastrulae. Note that *xTFG* transcripts do not show any dorso-ventral preference, unlike the organizer gene C*hordin* (*Chd*). *Histone 4* (*H4*) was used as a loading control and -RT as a negative control. (**J**, **K**) Whole mount *in situ* hybridization showing animal pole localization of *xTFG* maternal transcripts, at the 4-cell stage. **J** offers a top-view and **K** a side-view of the same embryo. (**L**-**N**) *xTFG* mRNA was expressed strongly in the cement gland (cg) at later stages of development (stage 20 and 26). Weaker staining could also be detected in the epidermis. (**O**) Bisected stage 26 embryos revealed specific *xTFG* mRNA staining in the notochord (no). Weaker expression was detected in the epidermis and the alar plate of the spinal cord. Scale bar represents 500 µm in **J**-**N**, and 200 µm in **O**.

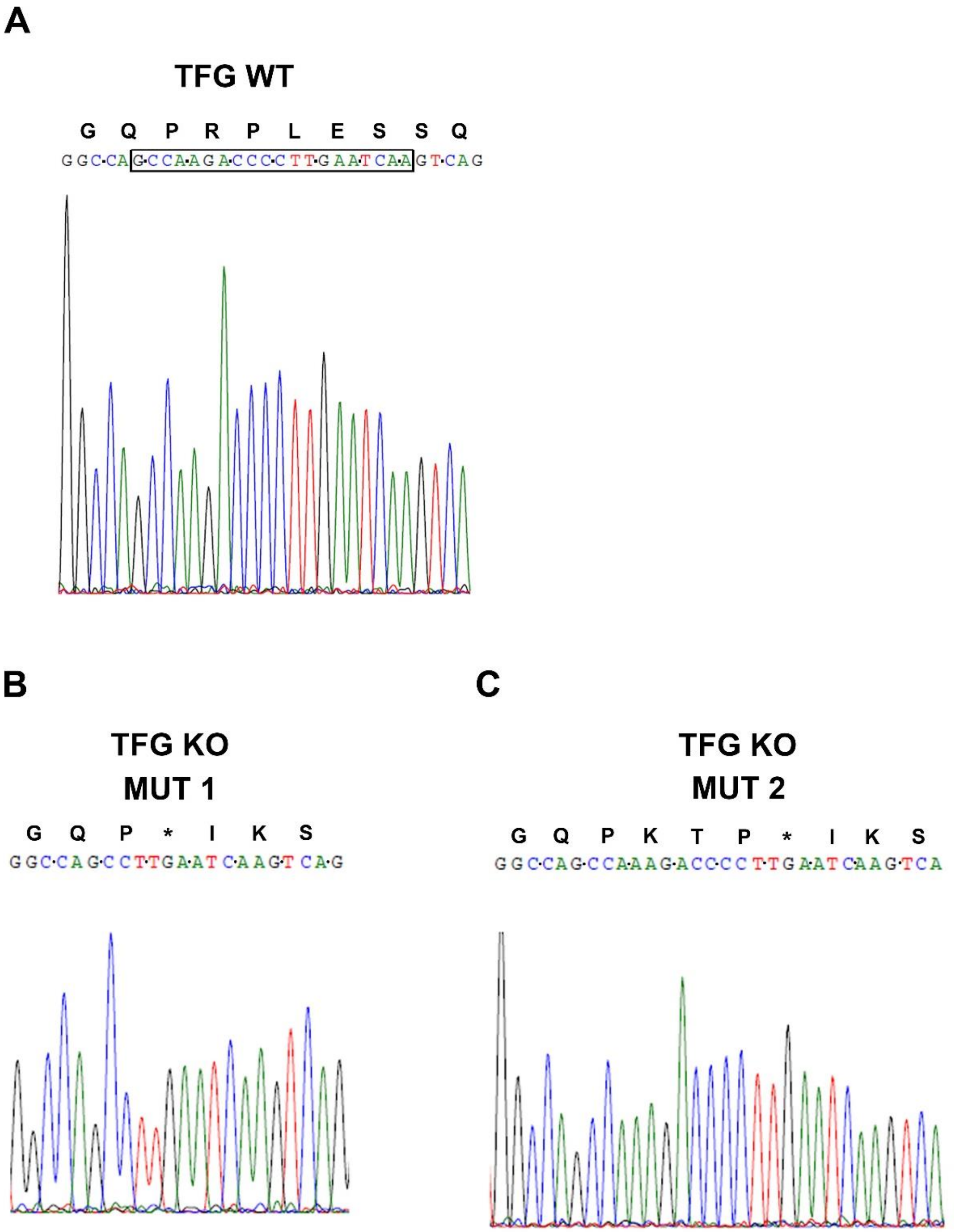
A
TFG WT
G Q P R P L E S S Q
GGC·CAG·CCA·AGA·CCC·CTT·GAA·TCA·AGT·CAG
B
TFG KO
MUT 1
G Q P * I K S
GGC·CAG·CCT·TGA·ATC·AAG·TCA·G
C
TFG KO
MUT 2
G Q P K T P * I K S
GGC·CAG·CCA·AAG·ACC·CCT·TGA·ATC·AAG·TCA

Fig. S5. TFG knock-out by CRISPR-Cas9 confirmed by genomic DNA sequencing. Genomic DNA was extracted from untransfected WT HEK293T cells, and from a cell clone stably transfected with a Cas9 expression vector containing an sgRNA-encoding sequence specific for human TFG. A 600 nucleotide region spanning the target sequence was amplified by PCR, cloned into sequencing vectors, and analyzed by Sanger sequencing. The reference chromatogram from sequencing results is shown below the DNA sequence. (**A**) Nucleotide sequence of TFG from wild-type (WT) HEK293 cells; the coding frame is indicated by black dots between each codon. The boxed area marks the target sequence recognized by the spacer region in the sgRNA in the WT TFG sequence. The predicted amino acid sequence is shown above the DNA sequence. (**B** and **C**) Sequences from a TFG knock-out clonal cell line showing the two types of mutant sequences resulting after PCR amplification of mutated genomic DNA. Each TFG allele harbored independent insertion-deletion (indel) events induced by Cas9 as a result of non-homologous end joining (NHEJ) repair. In Mutation 1 (Mut1) Cas9 induced a deletion of a short nucleotide sequence (8 nucleotides, AAGACCCC). Mutation 2 (Mut 2) resulted in the insertion of an extra adenine between the original CCA and AGA codons. Both indel events induced a frameshift mutation, introducing an early STOP codon (indicated by an asterisk), and generating truncated TFG mutant proteins of approximately 90 amino acids.

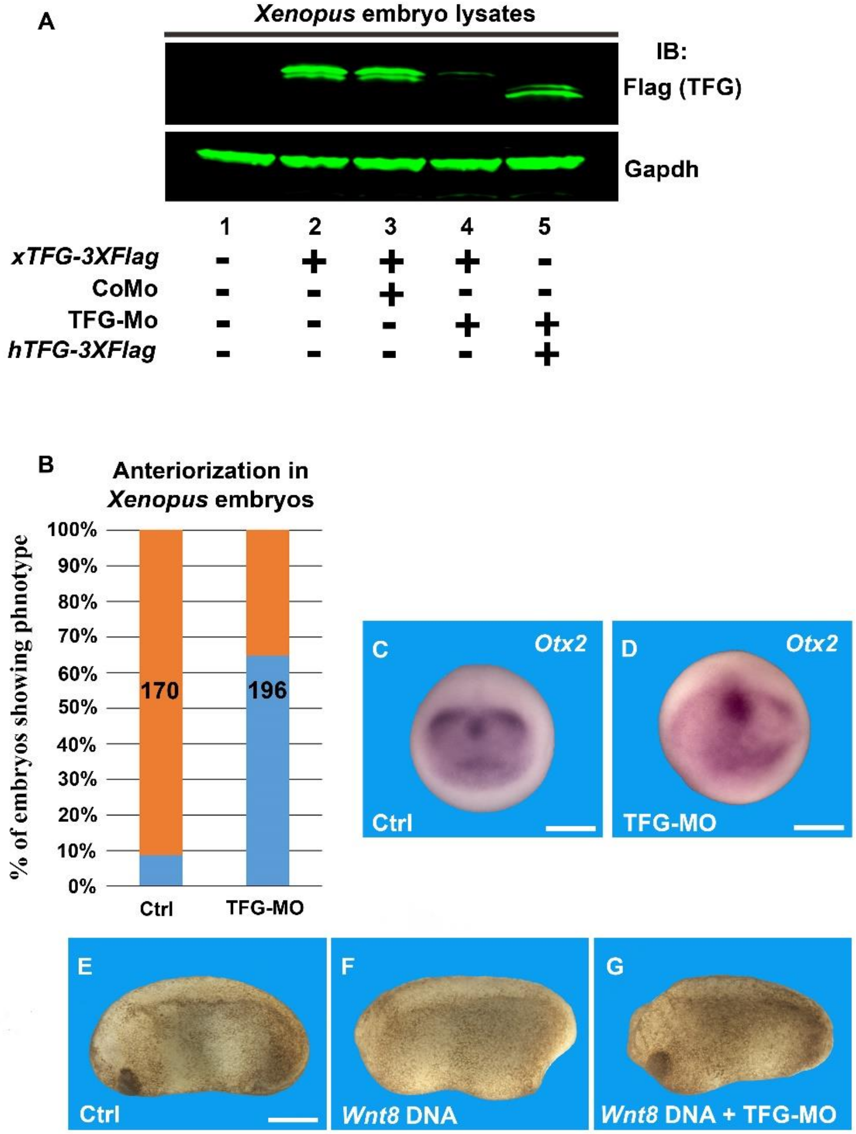

Fig. S6. TFG loss-of-function using a specific antisense Morpholino (MO) induces anteriorization, revealing a requirement for Wnt activity in *Xenopus* embryos. (**A**) TFG-MO is specific and blocked translation of Flag-tagged *Xenopus TFG* mRNA in microinjected embryos

(compare lanes 3 and 4), while mRNA encoding human *TFG* was not targeted by the morpholino (lane 5). Frog embryos were injected at 4-cell as indicated, cultured until gastrula stage and lysed for Western blot analysis. 400 pg/embryo of *Xenopus* or human Flag-tagged *TFG* mRNA were microinjected together with 72 ng/embryo of Control Morpholino (CoMO) or xTFG-MO. Uninjected embryos were used as a negative control (lane 1), and Gapdh used as loading control. (**B**) Quantification of the experiment shown in Fig. 5B and C of the main text. Numbers in the columns show the quantity of embryos used. TFG-MO induces anteriorization in over 60% of the injected embryos. (**C**, **D**) *In situ* hybridization for the anterior marker *Otx2*. Compared to control embryos (n=33), TFG-MO induced an expansion of *Otx2* in 65% of the injected embryos (n=20). (**E**-**G**) While control embryos showed normal antero-posterior patterning (n=103), but injection of *Wnt8* DNA induced loss of anterior development, as shown by the absence of the cement gland (90%, n=61). Co-injection of TFG-MO inhibited anterior truncation (65%, n=66), suggesting that Wnt signaling requires TFG. Scale bars in **C**-**D** represent 500 µm.

Blot Fig.1M

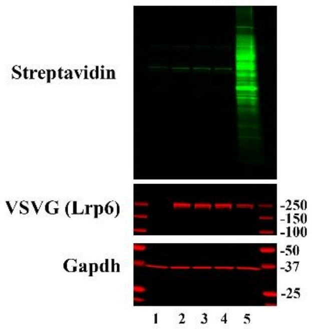

Blot Fig.4B

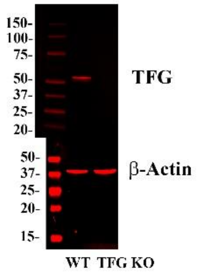

Blot Fig.S1I

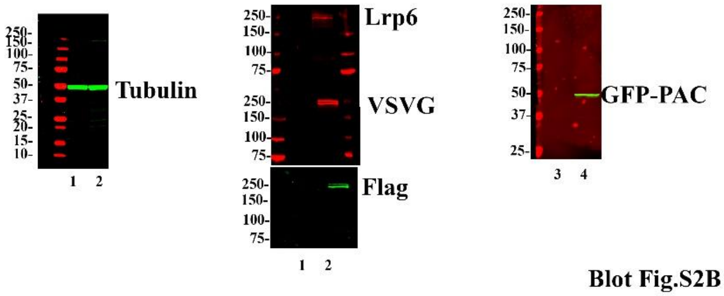

Blot Fig.S2A

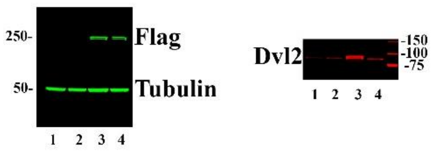

Blot Fig.S2B

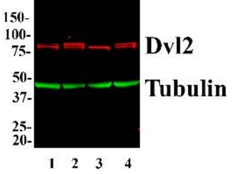

Blot Fig.S2C

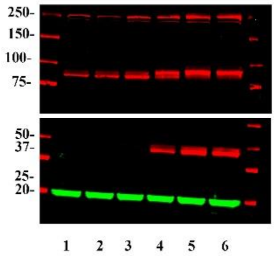

Blot Fig.S2E

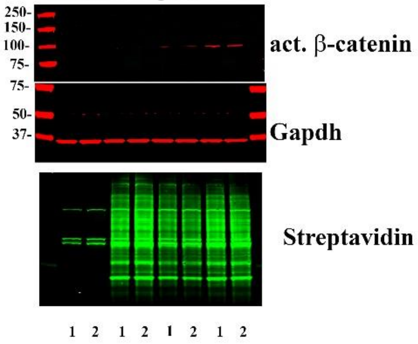

Blot Fig.S6A

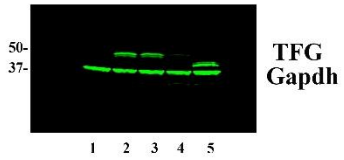

Agarose gel shown in Fig S4H,I

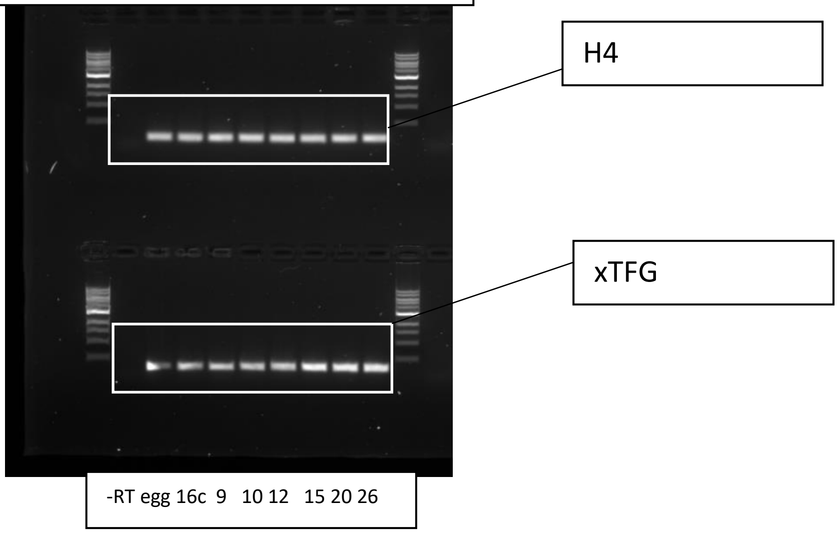

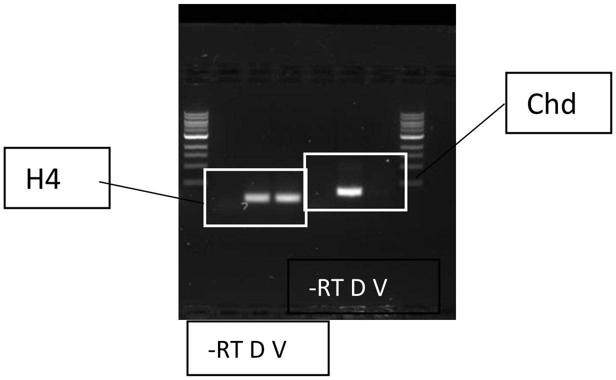

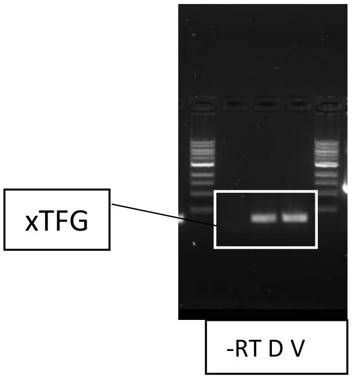

Supplementary Information File

Original scan images of the blots and agarose gels used in the indicated figures. Orientation is the same as in the figures.

Table S1. List of oligonucleotides used in this study.

Oligo Sequences		
**Use**	**Name**	**Sequence**
**Cloning (Gateway sequences in bold)**	xTFG Fw	**GGGGACAAGTTTGTACAAAAAAGCAGGCT**ACCATGAATGGACAACTGGACTTAAGCGG
	xTFG Rv	**GGGGACCACTTTGTACAAGAAAGCTGGGTC**TCGGTAACCAGGTCCTGGCTGAG
	hTFG Fw	**GGGGACAAGTTTGTACAAAAAAGCAGGCT**ACCATGAACGGACAGTTGGATCTAAGTG
	hTFG Rv	**GGGGACCACTTTGTACAAGAAAGCTGGGTC**TCGATAACCAGGTCCAGGTTGGG
	Lrp6 Fw	**GGGGACAAGTTTGTACAAAAAAGCAGGCT**ACCATGGGGGCCGTCCTGAGGAG
	Lrp6 Rv	**GGGGACCACTTTGTACAAGAAAGCTGGGTC**GGAGGAGTCTGTACAGGGAGAGG
	xNotum Fw	**GGGGACAAGTTTGTACAAAAAAGCAGGCT**ACCATGGCTGGGACCCTATGTGTAACC
	xNotum Rv	**GGGGACCACTTTGTACAAGAAAGCTGGGTC**ACTGCTTAACACACCAAGAAGTTTGC
	Apex2 Fw (XhoI)	CCGCTCGAGATGGACTACAAGGATGACGACGATAAG
	Apex2 Rv (XbaI)	CCGTCTAGATTAGTCCAGGGTCAGGCGCTCC
	Ires-GFP-PAC (XbaI)	CCGTCTAGACTCAAGCTTCGAATTCTGC
	Ires-GFP-PAC (SnaBI)	CCGTCTACGTATCAGTTATCCAGATCCGG
**CRISPR/Cas9**	hTFG sgRNA oligo Fw (BbsI)	CACCGTTGATTCAAGGGGTCTTGGC
	hTFG sgRNA oligo Rv (BbsI)	AAACGCCAAGACCCCTTGAATCAAC
	hTFG genomic DNA (Topo TA cloning) Fw	GGCATCAAGAAAGACTGATAATTCTGC
	hTFG genomic DNA (Topo TA cloning) Rv	GCTCCAGTAGGTCTATGATGAGATCC
**RT-PCR**	xTFG Fw	GATGAAGATGGAGATCTTATAACAATATTTG
	xTFG Rv	GGCAGCCATAACTTGGGTAGATG
	H4 Fw	CGGGATAACATTCAGGGTATCACT
	H4 Rv	ATCCATGGCGGTAACTGTCTTCCT
	Chd Fw	GTTGTACATTTGGTGGGAA
	Chd Rv	ACTCAGATAAGAGCGATCA
**Probe**	xTFG Fw	ATGAATGGACAACTGGACTTAAGCGG
	xTFG Rv (**T3 promoter** in bold)	**AATTAACCCTCACTAAAG**TCGGTAACCAGGTCCTGGCTGAG
**Knock-down**	TFG Morpholino	TTAAGTCCAGTTGTCCATTCATTGT
	BH Morpholino L	ATATCCCGAGCCAAACTGTAGCCAT
	BH Morpholino S	ATCCAGAGCCAAACTGTACCCATCT
	Lrp6 phosphorothioate (*) antisense DNA oligo	T*C*G*AGGCTGATCCAG*C*T*C

Table S2. List of primary and secondary antibodies used for Western Blot in this study.

**Western Blot**			
**Primary Antibody**	**Vendor**	**Catalog Number**	**Dilution**
Rabbit anti-VSV-G	Genetex	GTX18612	1:1000
Mouse M2 anti-Flag	Sigma	F3165	1:1000
Rabbit anti-Lrp6	Cell Signaling Technology	C5C7	1:1000
Rabbit anti-phospho-Threonine 1479 Lrp6	Abnova	PAB12632	1:1000
Rabbit anti-TFG	Invitrogen	MA5-32375	1:1000
Mouse anti-α-tubulin	Calbiochem	CP06	1:3000
Chicken anti-GFP	Aves Labs	1020	1:1000
Rabbit anti-Gapdh	Cell Signaling Technology	14C10	1:3000
Rabbit anti-Dvl2	Cell Signaling Technology	30D2	1:1000
Rabbit anti-active (non-phosphorylated) β-Catenin	Cell Signaling Technology	D13A1	1:1000
Rabbit anti-β Actin	Cell Signaling Technology	13E5	1:3000
Streptavidin IRDye 800 CW	Li-COR	926-32230	1:3000
**Secondary Antibody**			
Donkey anti-rabbit IRDye 680 RD	Li-COR	926-68073	1:3000
Donkey anti-mouse IRDye 800 CW	Li-COR	926-32212	1:3000
Donkey anti-chicken IRDye 680 RD	Li-COR	925-68075	1:3000

Table S3. List of primary and secondary antibodies used for Immunofluorescence in this study.

	**Immunoflorescence**		
**Primary Antibody**	**Vendor**	**Catalog Number**	**Dilution**
Mouse M2 anti-Flag	Sigma	F3165	1:300
Mouse anti-TFG	Invitrogen	MA5-25759	1:200
Mouse anti-β catenin	Santa cruz Biotech	sc-7963	1:200
Chicken anti-GFP	Aves Labs	1020	1:300
Rabbit anti-Hrs	Santa Cruz Biotechnology	M-79	1:100
Streptavidin Alexa Fluor 594	Invitrogen	S11227	1:1000
Phalloidin Alexa Fluor Plus 405	Invitrogen	A30104	1:1000
**Secondary Antibody**			
Donkey anti-mouse Alexa Fluor 488	Jackson ImmunoResearch Laboratories	715-546-150	1:1000
Donkey anti-rabbit Cy3	Jackson ImmunoResearch Laboratories	711-166-152	1:1000
Goat anti-chicken Alexa Fluor 488	Jackson ImmunoResearch Laboratories	103-545-155	1:1000
Donkey anti-mouse Cy3	Jackson ImmunoResearch Laboratories	715-166-150	1:1000

Supplementary Movie S1

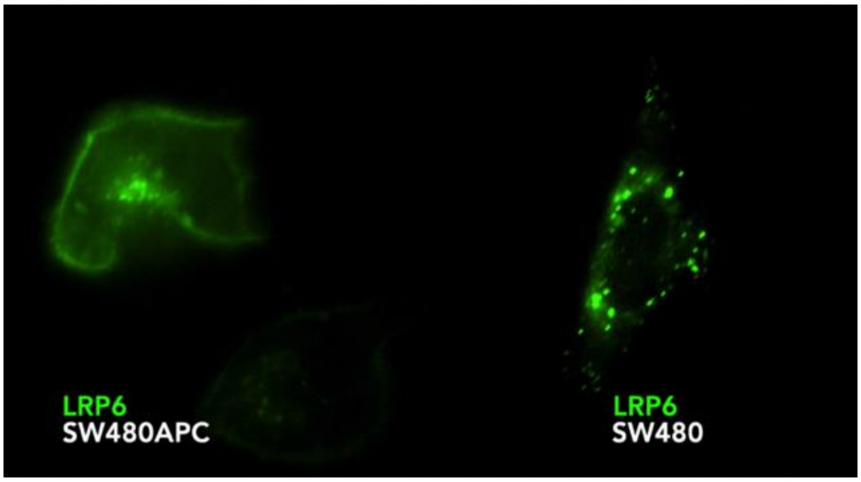

Supplementary Movie S1. Lrp6 is constitutively endocytosed in SW480 colon cancer cells in which Wnt signaling is activated by mutation of Adenomatous Polyposis Coli, the tumor suppressor initially mutated in 85% of colon cancers, but not in cells in which APC is restored. SW480 cells were transfected with *pCS2-Lrp6-eGFP* DNA. Green fluorescence filter with Apotome.2 optical sectioning was used to collect images of Lrp6-eGFP. Note that in absence of APC (SW480 cell, right side) Lrp6-eGFP is found predominantly in intracellular puncta which represent endolysosomes. In SW480 cells in which APC has been restored Lrp6-eGFP localizes to the plasma membrane (SW480APC, left side). The only difference between these two cells is the presence or absence of APC. Wnt activation by loss-of-function of APC has profound effects on Lrp6 endocytic trafficking.

Supplementary Methods

APEX2-mediated proximity labeling. Lrp6-APEX2 HEK293T stable clonal cells cells were seeded in T225 flasks (Thermo Fisher Sci.) and cultured in filter-sterile growth medium (DMEM supplemented with 10% Fetal Bovine Serum (FBS), 1% penicillin/streptomycin and 1% glutamine), at 37 °C and 5% CO_2. Importantly, the Porcupine inhibitor IWP-2 was also added to the medium, 2 µM final concentration, to prevent activation of Wnt signaling by ligands produced endogenously. When cells reached confluency, culture medium was replaced with 25 ml of growth medium supplemented with 500 µM Biotin-Phenol (BP, Iris Biotech). BP was omitted in the no-biotinylation (NoBio) control. For the Mock control samples, the medium was replaced again after 10 min with control conditioned medium (derived from control mouse L cells, ATCC) containing 500 µM BP and cells were incubated for an additional 20 min. For the Wnt treatment samples, BP-containing medium was replaced after either 10 min or 25 min with Wnt3a conditioned medium (derived from mouse L Wnt3a cells, ATCC) containing 500 µM BP, and cells incubated for an additional 20 min or 5 min, respectively. Thus, all samples were incubated with BP for a total of 30 min at 37°C. To start the biotin-labeling reaction, H_2O_2 was added directly to the BP-containing medium to a 1 mM final concentration 1 minute before the end of incubation and flasks were gently agitated. At the end of the 1-minute labeling reaction, cells were immediately washed 5 times with quencher solution (ice-cold Dulbecco's phosphate-buffered saline, DPBS, containing 10 mM sodium azide, 10 mM sodium ascorbate and 5 mM Trolox). To minimize cell detachment during washes, flasks were gently inverted to pour the wash solution on the bottom face, and then inverted again to cover cells with fresh solution. Finally, cells were vigorously washed off the flasks, pelleted by brief centrifugation, and lysed in 1.5 ml of RIPA lysis buffer (50 mM Tris, 150 mM NaCl, 0.1% wt/vol SDS, 0.5% sodium deoxycholate, 1% vol/vol Triton X-100 in nanopure water, pH 7.5, supplemented with 1x Roche cOmplete protease inhibitor cocktail, 10 mM sodium azide, 10 mM sodium ascorbate and 5 mM Trolox). Two T225 flasks were used for each sample, and experiments were conducted in duplicates.

Streptavidin pull-down. Cell lysates were cleared by centrifugation at 16,000 g for 10 min at 4ºC. Supernatants were transferred to new 1.5 ml Eppendorf tubes and protein concentration of each sample was determined using the Pierce 660-nm protein assay, following manufacturer's instructions. Streptavidin-coated magnetic beads (Pierce) were washed twice with RIPA buffer, and 2,000 µg of each protein lysate sample were then incubated with 100 µl of the magnetic bead slurry, on an end over end rotator for 1 hour at room temperature. Subsequently, the beads were washed twice with RIPA buffer, and an additional three times with RIPA buffer containing no Triton X-100/sodium deoxycholate since they interfere with subsequent mass spectrometry analysis, once with 1 M KCl, once with 0.1 M Na_2CO_3 pH 11, and three times with 8 M urea buffer in 100 mM Tris-HCl, pH 8.5. At this point, affinity-purified biotinylated protein samples were processed for further analysis by mass spectrometry.

Generation of TFG knock-out cell lines by CRISPR-Cas9 genome editing. A plasmid based on the original PX459 from Zhang lab, containing Streptococcus pyogenes Cas9 (spCas9)-2A-blasticidin resistance was obtained from Addgene (#118055, from Ken-Ichi Takemaru lab). The human TFG genomic sequence was analyzed with the CRISPR Design tool available online at Synthego.com, to identify potential target sequences followed by the NGG protospacer adjacent motif (PAM) sequence. A promising 20-nt sequence on the reverse, non-coding strand was

identified (5’-TTGATTCAAGGGGTCTTGGCTGG-3’, underlining indicates the PAM sequence) less than 300 nucleotides away from the TFG start codon. Oligos from both strands of the spacer TFG-specific sequence were annealed and phosphorylated following the directions from the Zhang lab protocol available at Addgene, https://www.addgene.org/crispr/zhang/. The double-stranded oligos were then cloned into spCas9-2A-blast, previously linearized with BbsI restriction enzyme (New England Biolabs). After transformation, plasmid DNA was extracted from bacterial colonies and analyzed by DNA sequencing for the presence of the sgRNA-encoding sequence. The resulting TFG-sgRNA spCas9-2A-blast plasmid was transfected with lipofectamine 2000 (Invitrogen) into HEK293T cells permanently transfected a Wnt-inducible β-catenin activated (BAR) Luciferase reporter and Renilla for normalization (see below). After 48 hours of transfection, permanently transfected cells were selected in medium containing 5 µg/ml blasticidin. Clonal lines were obtained by limiting dilution, expanded, and analyzed by western blot for TFG protein expression. One clone showed complete loss of TFG protein expression. To further confirm TFG gene KO, genomic DNA was extracted from mutant and WT cells and used as a template for PCR, using a proof-reading polymerase (Phusion high fidelity DNA polymerase, New England Biolabs). An amplification product of approximately 600 bp surrounding the Cas9 target sequence was obtained and ‘A-tailed’ (to add single A nucleotides at each 3’ end) using a standard non proof-reading taq polymerase (Taq 2X master mix, NEB). The tailed PCR product was then cloned into TOPO-TA cloning vector (Invitrogen), according to manufacturer instructions. Following transformation of the ligation reaction, multiple bacterial colonies were selected, grown, and plasmid DNA extracted and processed for sequencing.

Embryo manipulations. *Xenopus laevis* frogs were purchased from Nasco. A sperm suspension was obtained after crushing 1 whole testis in 1 ml of 1x Marc’s modified ringers solution (MMR, 0.1 M NaCl, 2.0 mM KCl, 1 mM MgSO4, 2 mM CaCl2, 5 mM HEPES, pH 7.4). Eggs were spawn in high salt solution (1.2x MMR) from female frogs injected the night before with 800 units of human chorionic gonadotropin (HCG, Lee BioSolutions). Collected eggs were fertilized in vitro using the sperm suspension described above and the developing embryos were cultured in 0.1x MMR and staged according to Nieuwkoop and Faber [8]. For *in vitro* mRNA synthesis, plasmid DNA from pCS2-hTFG-3xFlag, pCS2-xTFG-3xFlag, pCS2-xDkk1, pCS2-nLacZ, pCS2-Lrp6-APEX2 and pCS2-xNotum were linearized with NotI and transcribed with SP6 RNA polymerase using an Ambion mMessage mMachine kit. The amount of mRNAs injected per embryo is indicated in the figure legends. For knock-down experiments, a TFG translation-blocking Morpholino (MO) antisense sequence recognizing both *Xenopus* homeolog alleles was designed and synthesized by Gene Tools: 5’ TTAAGTCCAGTTGTCCATTCATTGT 3’. 36 ng of TFG MO (1:1 dilution of the 1 mM stock solution) were injected per embryo into the 2 dorsal animal blastomeres at the 4 to 8 cell stage. Injection at the 8-cell stage resulted in a stronger phenotype. For Sox2 staining and the *pCSKA-xWnt8* DNA injection experiment, embryos were injected 4 times (72 ng in total of TFG-MO) at the 2-cell (Sox2) or 4-cell stage (xWnt8 DNA, 32 pg total). A mixture of Bighead.L and Bighead.S MO (32 ng per embryo) was injected two times into the two dorsal blastomeres at the 4-cell stage, with or without TFG MO. Maternal knock-down of xLrp6 and host transfer of Lrp6-depleted oocytes was performed as previously described, using albino female frogs as hosts [9]. LacZ lineage tracing and *in situ* hybridization using antisense probes for *Sox2*, *Engrailed-2*, *Krox20*, *TFG*, *Otx2*, *Rx2a* and *MyoD* were performed according to standard protocols (http://www.hhmi.ucla.edu/derobertis/). The *Xenopus* TFG *in situ* hybridization probe was generated by PCR using a reverse primer containing a T3 promoter (Table S1). The

PCR product was then purified with a PureLink purification kit (Invitrogen) and used as a template to synthesize the antisense probe using the T3 promoter, as previously described [10].

Western Blots. Standard protocols were used to perform sodium dodecyl sulfate polyacrylamide gel electrophoresis (SDS-PAGE) and western Blot. A list of primary and secondary antibodies used in this study, including their dilutions, is provided in Table S2. Nitrocellulose membranes were blocked in TBST-milk 5%. Primary and secondary antibodies were diluted in TBST-milk 2.5%. For pLrp6 (Tp1479) western blot, nitrocellulose membranes were blocked with Li-COR blocking buffer (TBS-based) and Tp1479 antibody was diluted in the same blocking buffer supplemented with 0.2% Tween-20. Li-COR Odyssey 3.0 software was used to analyze the blotted membranes.

Heatmaps. Heatmaps were generated in R-Studio (1.2.1335) [11]. For Fig. 2C and Fig. S2F, normalized enrichment values of Wnt3a treated samples were used as inputs. Horizontal row z-scores were obtained from the values by calculating the mean, variance and standard deviation of each protein by using the ggplots v3.0.1 package in R-Studio [12]. The rows/proteins were left unclustered, as were the columns/treatments. The heatmap script used to generate our heatmaps was as follows:

```
##: Load gplots package
library(gplots)
##: Import Wnt dataset
Normalized.heatmap.of.Wnt.targets. <- read.csv("~/Desktop/APEX2 normalized data/Normalized heatmap of Wnt targets .csv", row.names=1)
##: Convert data into a matrix frame
Normalized.heatmap.of.Wnt.targets._matrix <- data.matrix(normalized.heatmap.of.Wnt.targets.)
## Generate heatmap via heatmap.2
heatmap.2(Normalized.heatmap.of.Wnt.targets._matrix, trace = "none", scale = "row", col = greenred(90), cexCol = 1, Colv = FALSE, Rowv = FALSE)
```

Immunofluorescence. HeLa cells were grown on 12-well plates containing glass coverslips. For HEK293T cell immunostaining, coverslips were pre-coated with a solution containing 0.01% poly-L-Ornithine (Millipore) overnight at 37°C. Two days following DNA transfection, the cell culture medium was replaced with control or Wnt3a conditioned medium as required, and cultured as indicated in the figure legends at 37°C. Cells were washed twice with PBS (phosphate buffered saline, 137 mM NaCl, 2.7 mM KCl, 10 mM Na_2HPO_4, 1.8 mM KH_2PO_4), fixed in 4% (wt/vol) paraformaldehyde (Sigma, P6148) in PBS for 20 min, then permeabilized with 0.2% (vol/vol) Triton X-100 in PBS. Note that for cell-surface staining of Lrp6 in some experiments we used a lower concentration of Triton X-100 (0.05%), which markedly improved plasma membrane immunofluorescence (e.g., Fig. 1*F*). Coverslips were then washed with PBS, blocked for 1 hour in blocking buffer consisting of 3% (wt/vol) BSA in PBS at room temperature, and incubated with primary antibodies in blocking buffer overnight at 4°C. The next day cells were washed 3 times with PBS, incubated with secondary antibodies diluted in blocking buffer for 1 hour at room temperature and mounted onto glass slides with ProLong Gold antifade reagent with DAPI (Life Technologies) to stain cell nuclei. A list of primary and secondary antibodies used in this study, including their dilution, is provided in Table S3. Images were acquired using a Zeiss Imager Z.1 microscope with Apotome and 63X oil immersion objective. Image analysis was achieved with Zeiss (Zen) and ImageJ (NIH) imaging software.

Image Quantification and Statistical Analyses. For quantitatively analyzing the Wnt3a-dependent increase in Hrs-containing vesicles, Hrs immunofluorescence was quantified in control and Wnt3a-treated cells using ImageJ software analyses (at least n=10 cells per condition). Briefly, this included normalizing fluorescence in images and measuring fluorescence in individual cells. Pearson's correlation coefficients were calculated using ImageJ software to assess the degree of colocalization between puncta in two different channels. Two-tailed t tests were used for two-sided comparisons, and $*P < 0.05$, $**P < 0.01$, and $***P < 0.001$ were considered to be statistically significant.

Movies. Time-lapse movies were acquired with a Zeiss Observer.Z1 microscope equipped with Apotome.2. The microscope has a fully automated stage and a Temperature/CO_2 Module S for cell culture. Images were collected using Colibri LED using green fluorescence filters with Apotome and a 63x LD (Long working Distance) Apochromat objective. Cells were grown on 4-well Nunc Lab-Tek II Chamber Slide treated with Fibronectin. Each chamber was incubated with 0.1 ml of 100 µg/ml of sterile Fibronectin (Sigma) for 30 min at room temperature, and washed 3 times with PBS before seeding cells in DMEM medium containing 10% Fetal Bovine Serum. Image acquisition was 40 frames in 20 min. Fluorescence filters were controlled by Axiovision 4.8 software and saved in this program. Movies were then processed using Adobe Premiere Pro CC 2019.

Luciferase assays. To test Wnt3a conditioned medium (Fig. S1H and Fig. S2D), a HEK293T cell line permanently transfected with β-Catenin-activated (BAR) reporter [13] Luciferase/Renilla system was used. Cells were plated in 12-well culture plates and incubated until the next day (at 60% of confluency) with either control or Wnt3a conditioned medium for 16 hours. After treatment, cells were lysed in 100 µl of Passive Lysis Buffer (Promega). Luciferase assays were performed with the Dual-Luciferase Reporter Assay System (Promega) using a Glomax Luminometer (Promega) according to manufacturer's instructions. Renilla readings were used for normalization. For the siRNA knock-down experiment shown in Fig. 3*I*, the following procedure was adopted. On day one, 2 million HEK-293T cells were plated in 6-well culture dishes. On day two, cells were transfected with either siRNA targeting TFG (Dharmacon) or Scrambled sequences, using BioT transfection reagent, in triplicate. On day three, cells were re-plated onto 12-well plates. On day four, cells were transfected with BAR-Luc Wnt reporter DNA, pCS2+ Renilla and pCS2+ carrier DNA, with each well receiving a total of 2 µg of DNA. The following DNA amounts were used: 1.2 µg BAR-Luc reporter, 0.4 µg pCS2+ Renilla, and 0.4 µg of pCS2+ empty vector. 24 hours following DNA transfection, cells were incubated with either Wnt3a or control medium for 16 hours, and Wnt activation analyzed by Luciferase assays as described above. For the luciferase assays shown in Figure 4, WT or TFG KO HEK293T BR (BAR/Renilla) reporter cells were treated with control or Wnt3a conditioned medium, or CHIR99021 5 µM final concentration, for 16 hours at 37 °C. Cells were then lysed and analyzed by luciferase assay as described above.

RT-PCR on *Xenopus* cDNA. *Xenopus* embryos were collected at the desired developmental stages, and total RNA was extracted from groups of 3 embryos each with the RNeasy Mini Kit (QIAGEN) following manufacturer's instructions. 350 µl of lysis buffer were used per group of embryos, and on-column DNAse treatment was performed to remove genomic DNA following the Qiagen protocol. 2 µg of total RNA was used for cDNA synthesis with random hexamer primers,

using the AffinityScript Multi-Temp Reverse Transcriptase (Agilent). cDNA was then used for semiquantitative RT-PCR, and the PCR products were resolved on 0.8% Agarose gel. Primer sequences are listed in Table 1.

Supplementary References

1. Kirsch, N. *et al*. Angiopoietin-like 4 is a Wnt signaling antagonist that promotes LRP6 turnover. *Dev. Cell* **43**, 71-82 (2017).

2. Kim, K.A. *et al*. R-Spondin family members regulate the Wnt pathway by a common mechanism. *Mol. Biol. Cell* **19**, 2588-2596 (2008).

3. Guger, K.A. & Gumbiner, B.M. A mode of regulation of beta-catenin signaling activity in Xenopus embryos independent of its levels. *Dev. Biol.* **223**, 441-448 (2000).

4. Staal, F.J., van Noort, M., Strous, G.J. & Clevers, H.C. Wnt signals are transmitted through N-terminally dephosphorylated beta-catenin. *EMBO Rep.* **3**, 63-68 (2002).

5. van Noort, M., Meeldijk, J., van der Zee, R., Destree, O. & Clevers H. Wnt signaling controls the phosphorylation status of beta-catenin. *J. Biol. Chem.* **277**, 17901-17905 (2002).

6. Zhou, Y. *et al.* Metascape provides a biologist-oriented resource for the analysis of systems-level datasets. *Nat Commun.* **10,** 1523 (2019).

7. Slosarek, E.L. *et al.*, Pathogenic TFG mutations underlying hereditary spastic paraplegia impair secretory protein trafficking and axon fasciculation. *Cell Rep.* **24**, 2248-2260 (2018).

8. Nieuwkoop, P.D. & Faber, J. *Normal Table of Xenopus laevis (Daudin): A Systematic and Chronological Survey of the Development from the Fertilized Egg till the End of Metamorphosis* (North-Holland Publishing Co., Amsterdam, 1967), reprinted in 1994 (Garland Publishing, Inc., New York).

9. Colozza, G. & De Robertis, E.M. Maternal syntabulin is required for dorsal axis formation and is a germ plasm component in Xenopus. *Differentiation* **88**, 17-26 (2014).

10. Suzuki, T., Akimoto, M., Mandai, M., Takahashi, M. & Yoshimura, N. A new PCR-based approach for the preparation of RNA probe. *J. Biochem. Biophys. Methods* **62**, 251-258 (2005).

11. R Core Team. R: A language and environment for statistical computing. R Foundation for Statistical Computing, Vienna, Austria. URL http://www.R-project.org/ (2014).

12. Wickham, H. ggplot2: elegant graphics for data analysis. Springer New York (2009).

13. Biechele, T.L. & Moon, R.T. Assaying beta-catenin/TCF transcription with beta-catenin/TCF transcription-based reporter constructs. *Methods Mol. Biol.* **468**, 99-110 (2008).
